# Spatial transcriptomics reveals distinct cell type dynamics following opioid dependence in mice with the common human variant in the μ-opioid receptor, *Oprm1* A118G

**DOI:** 10.21203/rs.3.rs-7199524/v1

**Published:** 2025-08-18

**Authors:** Yihan Xie, Omar Guessoum, Johnathan Schug, Adrienne Jo, D. Kacy Cullen, Klaus H. Kaestner, Julie A. Blendy

**Affiliations:** 1 Department of System Pharmacology and Translational Therapeutics, School of Engineering and Applied Science, University of Pennsylvania; 2 Department of Genetics, School of Engineering and Applied Science, University of Pennsylvania; 3 Department of Neurosurgery, Perelman School of Medicine, School of Engineering and Applied Science, University of Pennsylvania; 4 Department of Bioengineering, School of Engineering and Applied Science, University of Pennsylvania

## Abstract

Opioid Use Disorder (OUD) is a multifaceted neuropsychiatric disease that can arise from genetic, environmental, and neurobiological factors. The *OPRM1* A118G single nucleotide polymorphism (rs1799971) encodes an N40D variant in the μ-opioid receptor (MOR) and is linked to increased risk of opioid and other drug dependencies, though its exact mechanism remains unknown. With the ongoing opioid crisis driving record overdose deaths, understanding how this variant influences addiction risk could open new therapeutic avenues. We applied a systematic, cross-modality platform to assess cell dynamics using spatial transcriptomics and uncover not only distinct spatially resolved transcriptome changes in opioid exposed mice, but also changes dependent on the *Oprm1* variant. Collectively, our findings suggest that genetic risk for opioid dependence at the *Oprm1* locus may be reflected more strongly in glial cell adaptations rather than neuronal dysfunction, emphasizing the importance of oligodendrocyte-mediated neuroimmune interactions in opioid dependence.

## Introduction

Opioid Use Disorder (OUD) is a chronic relapsing brain disease, and opioid related deaths continue to be a major problem in the United States in terms of socioeconomic and health care burden. Repeated exposure to opioids leads to OUD by inducing long-lasting changes in the function of brain circuits. Genetic factors account for up to 80% of disease risk for opioid dependence^1,2^. The functional variant rs1799971 (A118G, encoding Asn40Asp) is one of the most extensively studied candidate variants for substance use traits^3–5^. Evidence suggests that this single-nucleotide polymorphism (SNP) may have several functional effects, including altering β-endorphin binding and activity^6^, influencing cortisol response to naloxone blockade^7^, affecting neurobehavioral functions in mice with the equivalent mouse models^8–10^, and modulating synaptic function in human-induced pluripotent stem cell lines^11^. However, our understanding of how this risk variant alters brain function, network dynamics, and structural adaptations at the transcriptional level remains limited.

Single-cell RNA sequencing and other transcriptomic approaches have uncovered significant molecular and cellular changes linked to opioid addiction, such as cell type-specific transcriptional responses, alterations in glial cells, and transcriptional signatures of brain circuits associated with drug intake and relapse^12–19^. While these studies highlight critical molecular changes, all lack the spatial resolution required to map regional specificity and cell-cell interactions within the opioid-dependent brain. Recently developed spatial transcriptomics technologies allow for the quantification of RNA molecules and their positional relationship across cell-types and provide insights unattainable with traditional transcriptomic techniques. To date, the spatial distribution of transcripts in substance use disorders has not been documented.

To address this knowledge gap, we leveraged spatial transcriptomics in mice with the equivalent *Oprm1* variant, A112G, to identify and map gene expression signatures in brains of mice homozygous for either the risk (G) or the protective (A) allele. Our goal is to identify region-and cell-type specific molecular changes associated with genetic variance and opioid dependence to help explain phenotypes associated with this SNP. To facilitate our analysis, we developed a cross-modality platform to assess the cell dynamics of spatial transcriptomics (CellDynamicsST). This platform allows for flexible cell registration, hierarchal mapping, customizable cell type annotation and visualization, and provides the analytical framework to study the underlying cell state dynamics and network connectivity. By mapping gene expression changes within the anatomical context of the mouse brain following opioid dependence, our integrative spatial transcriptomic analysis uncovers a fundamentally new understanding of how the *Oprm1* A118G variant shapes the brain’s molecular response to chronic opioids.

## Results

To identify the impact of genetic risk on the development of opioid dependence, we profiled mice homozygous for the protective A or risk G-allele of the *Oprm1* gene. Our previous work demonstrated that the *Oprm1* A112G SNP modulates brain network dynamics during the transition from initial opioid exposure to dependence, with opioid-dependent GG-females showing significantly less stable brain states compared to GG-males or AA mice of either sex^20^. This instability suggests a distinct sex by genotype transcriptional and cellular adaptations that remain unexplored at a spatially resolved level and justified our focus on female mice in the current study.

We collected over 1.05 million single-cell transcriptomes from four treatment groups: AA SAL (saline control), AA MOR (chronic escalating morphine), GG SAL (risk allele; saline control), and GG MOR (chronic escalating morphine). Our analysis encompassed 247 brain regions as defined by the Allen Mouse Brain Atlas (CCFv3) across hierarchical anatomical levels. This included 223 level-seven structures, 183 level-six structures, and 52 higher-order meso-structures. Given our focus on opioid-mediated neuronal responses, we prioritized six major meso-structures: the cerebral cortex (CTX), cerebral nuclei (CNU), interbrain (IB), midbrain (MB), hindbrain (HB), and cerebellum (CBX).

**Fig. 1:**
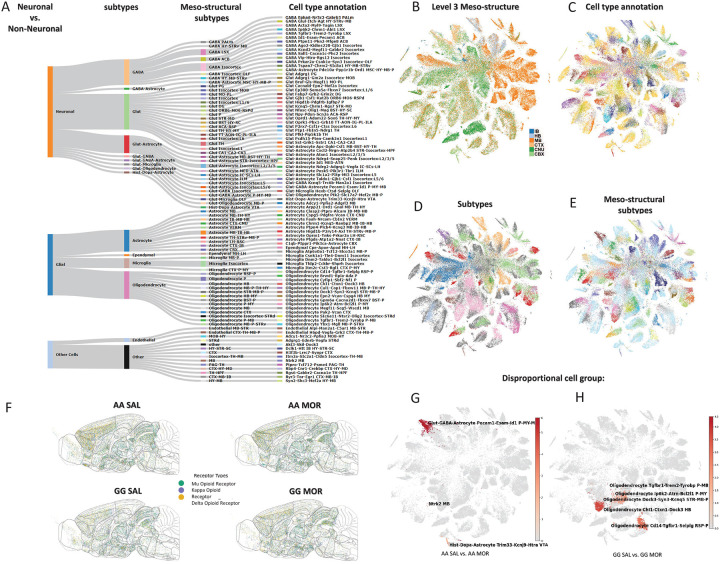
Transcriptomic Cell Type taxonomy of opioid naïve and dependent mouse brain. **(a)** A dendrogram of transcriptomic taxonomy tree of 92 classes (Cosmx SMI, n = 949,137 nuclei). Hierarchical annotation levels classify cell in top-down order (left to right): Neuronal or Non-neuronal, general subtypes of cells, meso-structural or regional specific cell subtypes, unique cell type or cell clusters. **(b - e)** t-SNE representation of cells colored by meso-structural composition **(b)** (based on Allen Mouse Reference Atlas, depth = 3) **(c)** unique cell clusters **(d)** major cell types **(e)** meso-structural cell types. **(f)** Representation of opioid receptor distribution in naïve vs. chronic opioid mice brain. **(g)** Cell clusters showed disproportional expression of chronic opioid specific Differential Expressed Genes (DEGs) in AA or **(h)** GG.

Using generalized clustering algorithms, we identified 137 unique cell clusters, hierarchically classified into four nested levels: neuronal vs. non-neuronal cell types (glial and other types), 19 subtypes, 57 meso-structural subtypes, and 137 cell types annotated based on marker gene expression, neurotransmitter composition, and tissue location (Fig. 1A–C). These clusters consolidated into 32 major cell types based on established marker genes^21^. Among 0.94 million cells passing quality control (see Methods), major populations annotated included 21.40% Oligodendrocytes and 4.32% Late Oligodendrocytes, 19.07% astrocytes, 17.23% Excitatory Neurons, 5.24% Inhibitory Neurons, 1.52% Basket Cells, 0.48% CCK Basket Cells, 0.09% Other Interneurons, 0.12% Dopaminergic Neuron, 5.70% Endothelial Cells, and 3.95% Microglia. These results provide a robust framework for examining transcriptional changes associated with genetic risk and opioid dependence. (Fig. 1D,E) (full summary including minor cell types is provided in Supplemental Data).

### Cell type Distribution in *Oprm1*AA and GG Opioid Dependent Mice

We mapped neuronal cell types in AA and GG mice under saline conditions. Cell types were identified by receptor and transporter expression profiles to characterize neurotransmitter-specific populations. Among all identified neurons, glutamatergic and GABAergic neurons were the most abundant (24.2% and 44.4%, respectively). Dopaminergic (2.1%), serotoninergic (3.1%), and noradrenergic (0.7%) neurons were relatively sparse, consistent with their known distributions in the mouse brain. Regionally, glutamatergic neurons were highly enriched in the isocortex, hippocampus, thalamus, and olfactory areas, while GABAergic neurons were more prominent in the striatum and hypothalamus. Dopaminergic cells were concentrated in the ventral tegmental area (VTA), whereas serotoninergic neurons were primarily located in the midbrain raphe nuclei. These data are consistent with cell type distribution documented in the literature.

Of interest, the spatial distribution and density of opioid receptor-expressing cells differed both by genotype and opioid exposure (Fig. 1F). Across all groups and brain regions, we observed 14.4% *Oprm1* (mu-opioid receptor), 10.8% *Oprk1* (kappa-opioid receptor), and 7.9% *Oprd1* (delta-opioid receptor) expressing cells. These proportions align with previous reports estimating that the various opioid receptor-expressing cells together comprise 20–30% of total brain cells^22^. Notably, in the opioid-naïve state, the frequency of *Oprm1* expressing neurons was lower in GG mice compared to AA mice (AA: 16.0%; GG: 12.7%), consistent with prior mRNA and protein analyses^8,23^.

### Opioid Dependent Transcriptome Differs by Genotype

Next, we identified marker genes with enriched expression in any of the 137 cell types in our atlas (P < 0.05, log2 absolute |FC| > 1 in a given cell type versus all others). The genes defining cell types were frequently associated with key neuronal processes, including neurotransmitter signaling, synaptic function, cytoskeletal organization, neuroprotection, and neurodegeneration **(Supplemental Table 1-DEG list)**. Among these, the following well-established markers of synaptic modulation were expressed in a high percentage of cells, including *Pvalb* (12.6% of total cells), *Sst* (8.8%), Vip (4.6%), *Npy* (11.3%), and *Cck* (7.7%). Genes involved in inhibitory neuronal function, including *Gad1* (11.9%) and *Gad2* (11.6%), were highly expressed in over 70 distinct cell clusters, consistent with the widespread role of GABAergic signaling in the brain. Similarly, genes encoding glutamate receptors and transporters (*Gria1* 4.2%, *Slc17a6* 6.0%, *Slc17a7* 5.7%, *Slc32a1* 10.2%) were marking multiple cell types. Genes associated with dopaminergic and catecholaminergic systems, which play key roles in reward processing and mood regulation, were also expressed in specific cell clusters, including *Th* (5.3%) and *Slc18a2* (4.2%). Analysis of the most commonly expressed genes across multiple cell clusters revealed several markers associated with neuroadaptive changes. These included *Ndrg4* (14.3%), which is implicated in cell growth, survival, and stress response; *Atp1b1* (12.7%), an ATPase subunit critical for maintaining neuronal excitability; *Sncb* (11.1%) and *Snap25* (11.0%), both of which are key regulators of neurotransmitter release and synaptic plasticity; and *Nap1l5* (10.5%), a gene involved in neuronal plasticity and transcriptional regulation. Thus, spatial transcriptomics enabled us to define marker genes for multiple cell types in the mouse brain.

In order to determine which cell types exhibited the most differentially expressed genes (DEGs) following opioid exposure by genotype, we first normalized the number of upregulated and downregulated DEGs in both genotypes (*Oprm1* AA and GG). We calculated the DEG ratio for each cell type in each condition (AA SAL vs. AA MOR; GG SAL vs. GG MOR) and then scaled these ratios across all cell types to obtain z-scores. To quantify the extent of DEG expression within each cell type, we applied a threshold of ≥1 UMI in ≥10% of cells within a given cluster. To assess whether individual DEGs were significantly enriched or depleted in specific cell populations, we calculated scaled (z-scored) expression of all DEGs across the cell types defined above (Fig. 1G,H). In AA mice, we identified several neuronal cell type clusters that disproportionately responded to chronic opioid exposure compared to all other cell types. Among these, a key population of interest were dopaminergic neurons in the ventral tegmental area (VTA) with marker gene expression of *Trim33*, *Kcnj9*, and *Htra*. Trim33 is involved in transcriptional regulation in response to extracellular signals^24^, *Kcnj9* (also known as *Girk3*) modulates neuronal excitability and plasticity in response to drugs of abuse^25^, while *Htra* is important to maintain homeostasis during cellular stress^26^. Given the VTA’s central role in the reward circuitry, these transcriptional adaptations may reflect opioid-induced neuroplasticity mechanisms that drive dependence-related behaviors.

In striking contrast, cell types that showed the most differentially expressed genes in opioid-dependent *Oprm1* GG mice were glial cells, particularly oligodendrocytes. Multiple oligodendrocyte clusters were defined by a spectrum of cell state markers associated with neuro-modulatory response genes. One population of oligodendrocytes, located in the pons and medulla, is marked by expression of *Ip6k2* (encoding Inositol Hexaphosphate Kinase 2), overexpression of which sensitizes cells to multiple apoptotic stimuli^27^. Increased levels of *Atrn* (Attractin), in these oligodendrocytes, suggest activation of the innate immune response during inflammation^28^. In addition to this pro-inflammatory state, oligodendrocytes also exhibited increased expression of the anti-apoptotic gene *Bcl2l1* suggesting concurrent activation of cell survival mechanisms^29^. A separate cluster of oligodendrocytes are marked by *Trem2* (encoding Triggering Receptor Expressed On Myeloid Cells 2) and *Tyrobp* (encoding Transmembrane Immune Signaling Adaptor); both important for neuroimmune adaptation. Another oligodendrocyte population in the hindbrain displayed increased expression of two genes associated with neuronal structural integrity, *Chl1* and *Dock3*. *Chl1* (Cell Adhesion Molecule L1 Like) supports neuronal survival, myelination, and axonal growth and has been implicated in the regulation of apoptosis^30,31^. *Dock3* (Dedicator Of Cytokinesis 3) is primarily known for promoting axonal outgrowth via the BDNF pathway in neurons^32^, but its function in oligodendrocytes is less clear. A third oligodendrocyte cluster in the striatum and midbrain co-expressed *Dock3* and *Syn3*, a neuronal marker, suggesting potential oligodendrocyte-neuron interactions at the synaptic level. In addition, elevated expression of *Kcnq5*, a voltage-gated potassium channel which is expressed in both neurons and oligodendrocytes^33^, supports the notion that this cluster is important in these cell interactions.

An oligodendrocyte group specific to the retrosplenial cortex (RSP) was marked by *Cd14*, *Tgfbr1* (encoding the TGF-beta receptor 1), and *Selplg* (encoding the Selectin P ligand), genes indicative of a reactive state in response to neuroinflammation or injury. *Cd14* and *Selplg* suggest immune-modulatory roles that could involve microglia or other immune cells^34,35^, while *Tgfbr1* is directly involved in oligodendrocyte differentiation and myelination^36^. This expression profile points to oligodendrocytes playing a dual role in myelination and immune response in response to chronic opioid exposure. Conventional models of opioid addiction have focused on neuronal dysfunction; however, our findings suggest that genetic risk for opioid dependence at the *Oprm1* locus may be reflected more strongly in glial cell adaptations, underscoreing the importance of oligodendrocyte-mediated neuroimmune interactions in opioid dependence.

### Cell State Distribution at the Level of Meso-Structures

**Fig. 2:**
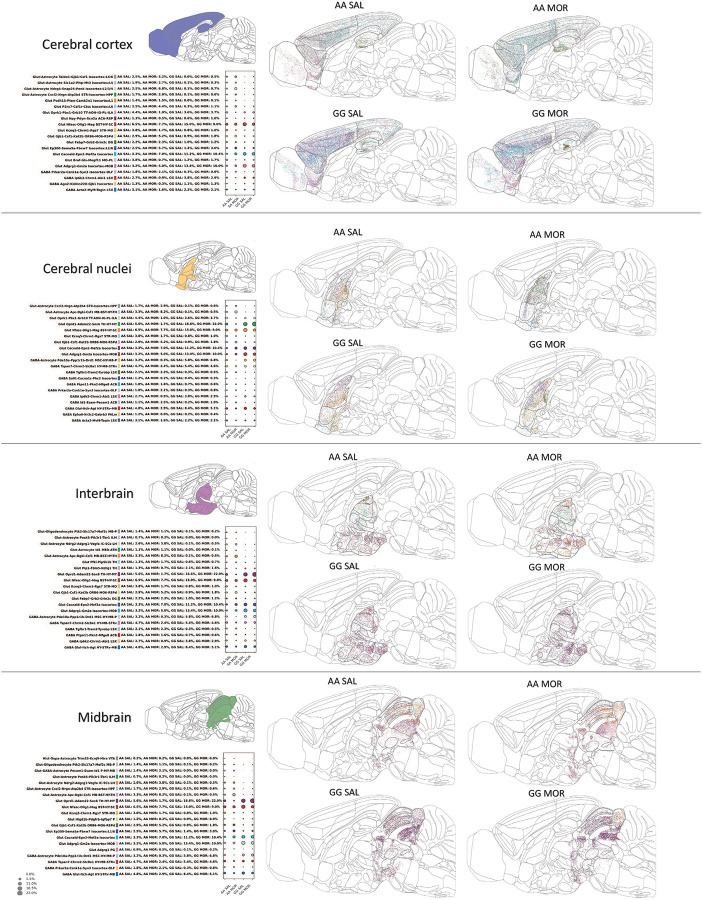
Atlas-based mapping of neuronal cell type distribution across meso-structural brain regions. Spatial distributions of neuronal cell types were visualized using the Allen Mouse Brain Atlas (CCFv3, hierarchical depth = 3), including four major meso-structures: the Cerebral Cortex (CTX), Cerebral Nuclei (CNU), Interbrain (IB), and Midbrain (MB). For each meso-structure, the top 20 most abundant neuronal cell types (based on relative frequency) were plotted for each experimental condition: AA SAL, AA MOR, GG SAL, and GG MOR (shown clockwise from top left). Cell distributions were projected and normalized to corresponding anatomical contours. Cell types are color-coded consistently across conditions. For each group, relative abundance was calculated and displayed as a percentage of total cells within that meso-structure. Dot plot summary of neuronal cell type composition. To aid comparative interpretation, a dot plot summarizes the relative abundance of each neuronal cell type across the four experimental conditions. The x-axis represents experimental groups; the y-axis lists the top 20 neuronal cell types aggregated across regions. Dot color matches the spatial plots, and dot size encodes the percentage of total cells per group. Color-legend matrix with group-wise percentages. An accompanying heatmap-style panel displays cell type–specific color assignments with their corresponding abundance across experimental groups. For each cell type (row), color swatches indicate the identity, while adjacent columns list the percentage contribution of that cell type to each experimental group (AA SAL, AA MOR, GG SAL, GG MOR). This concise format preserves spatial color encoding and facilitates side-by-side comparison of group-specific cell type compositions.

To identify possible genotype- and treatment-dependent changes in cell state distribution across brain regions, we next examined cell states at the meso-structural scale. We plotted the top 20 most abundant cell states in each meso-structure and compared their relative proportions between AA and GG mice (Fig 2). Overall, GG mice exhibited lower cell state diversity, while AA mice displayed a broader distribution of diverse cell states, particularly in the cerebral cortex, as well as interbrain, and midbrain (For detailed analysis of each mesostructure, see Supplemental Fig 2).

### Pathway and Functional Analysis

**Fig. 3:**
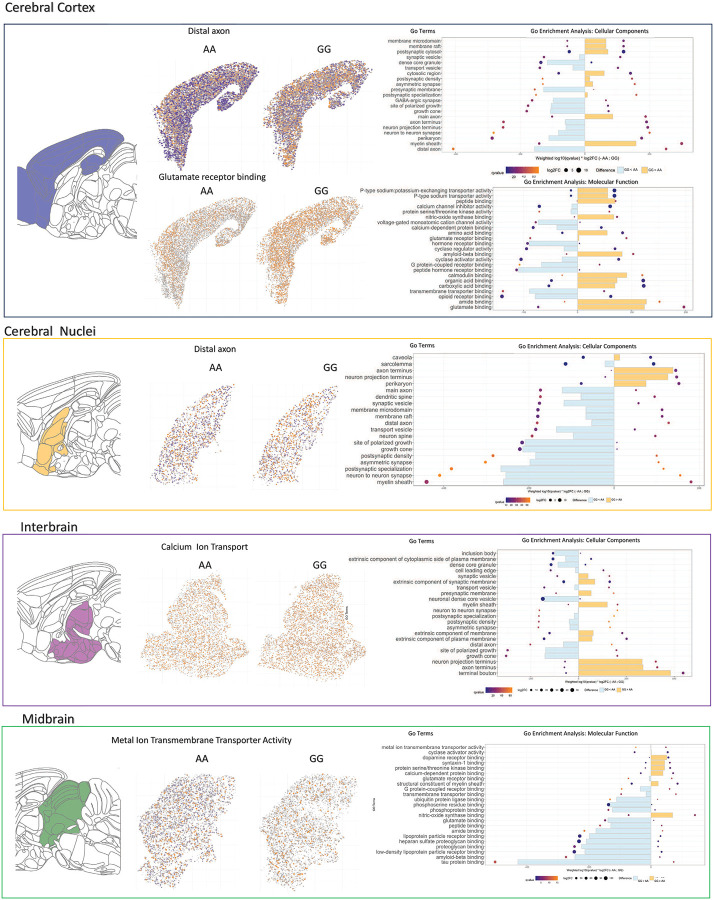
Molecular Function, Cellular Component, and Biological Function dynamics in opioid dependent brain state. GO Enrichment plot (right) showing top go categories enriched in opioid dependent brain state (over naïve state), dot size showing log fold change of gene set in each go category, dot color showing q value or significancy of each comparison, bar plot showing relative difference in GO enrichment score calculated by weighed log10(q value) * log2FC between AA and GG (orange = AA < GG, blue = AA > GG), selective categories are shown (full categories and data are available in supplemental). Spatial GO enrichment plot (left) showing cells that are enriched in AA or GG opioid dependent brain state (comparing opioid dependent over opioid naive) of selective go categories.

To further investigate the functional alterations underlying opioid dependence across brain meso-structures, we conducted pathway enrichment analysis on differentially expressed genes (DEGs) identified within each region. We examined enriched Gene Ontology (GO) terms for Biological Process, Molecular Function, and Cellular Component, comparing the top 20 functional categories between AA and GG mice following chronic opioid administration (Fig 3).

Our analysis revealed distinct patterns of pathway enrichment between AA and GG mice, particularly in the cerebral cortex. In AA mice, genes involved in distal axon function (GO: 0150034), G-protein coupled receptor binding (GO: 0001664), opioid receptor binding (GO: 0031628), and GABAergic synapse function (GO: 0098983) were significantly upregulated in the opioid-dependent state. By contrast, in GG mice amide binding (GO: 0033218) and glutamate binding (GO: 0035254) pathways were enriched in cortical regions, suggesting a shift in neurotransmission-related molecular adaptations in these mice.

To visualize the spatial distribution of functionally enriched cell populations, we mapped individual cells exhibiting significant upregulation of DEGs in representative GO categories across four brain regions. This revealed marked spatial differences in enriched functional pathways between AA and GG mice. In the cerebral cortex glutamate receptor binding was regionally enriched in the prelimbic (PL), infralimbic (ILA), and anterior cingulate cortex (ACA) regions of AA mice after chronic opioid exposure. However, GG mice showed a more widespread enrichment across the entire cerebral cortex (Fig 3, Cerebral Cortex), suggesting a genotype-dependent regional specificity in glutamatergic receptor function.

In the midbrain, GG mice displayed significantly less enrichment of metal ion transmembrane transport activity in the periaqueductal gray (PAG) and raphe nuclei (RN) compared to AA mice following chronic opioid exposure. Overall, across anterior-to-posterior brain regions, AA and GG mice exhibited differential enrichment patterns in a variety of GO categories that related to GABA-ergic signaling glutamate receptor binding, opioid-receptor binding calcium signaling as well as key biological processes impacted by opioid exposure like locomotor activity, sleep/wake and regulation of behavior (see full GO enrichment profiles in **Supplemental Table 1**).

### Spatially Resolved Gene Network and Pathway Remodeling in Opioid-Exposed AA and GG Brains

**Fig. 4:**
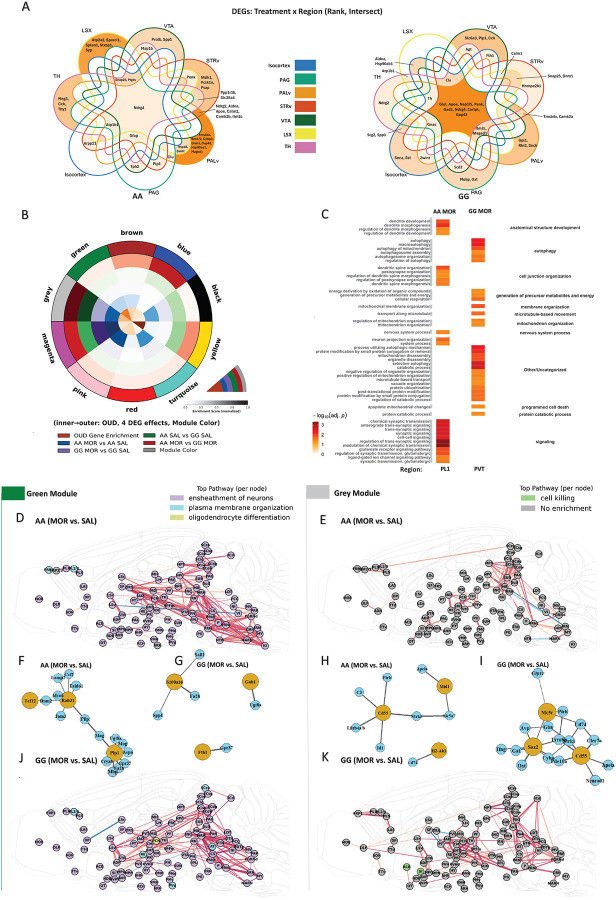
**(a) Venn diagram of inter-regional DEGs under chronic opioid exposure.** This Venn diagram displays the overlap of top-ranked treatment-sensitive DEGs across seven level 5 brain regions (LSX, VTA, STRv, PALv, PAG, Isocortex, TH). Genes were identified based on significant treatment-by-region interaction effects (padj < 0.01, |log_2_FC| > 1.5) and reflect changes in regional expression contrasts rather than bulk regulation. Multi-region overlaps highlight core genes involved in widespread spatial reorganization, while region-specific DEGs suggest localized disruption of anatomical identity. Each region intersection in the Venn diagram corresponds to genes differentially expressed in response to morphine in that region (or region combination) and not others. For example, in AA mice, *Pvalb* and *Spp1* are morphine-responsive only in VTA, while in GG mice, *Slc6a3* and *Cck* show VTA-specific upregulation. See main text and Supplemental Table 1 for comprehensive region/gene intersections. **(b) Consensus module prioritization and multidimensional enrichment visualization via circos plot.** Each segment around the circumference represents a consensus WGCNA gene coexpression module, colored according to WGCNA module color. For each module, concentric rings (inner to outer) encode the normalized −log_10_(p) enrichment for (1) opioid use disorder (OUD) risk genes, (2) differentially expressed gene (DEG) enrichment for AA MOR vs. AA SAL, (3) DEG enrichment for GG MOR vs. GG SAL, (4) DEG enrichment for AA vs. GG in the saline condition, and (5) DEG enrichment for AA vs. GG in the morphine condition. Enrichment values for each module and effect were computed by Fisher’s exact test, followed by normalization within each effect. Module color is shown in the outermost ring. This plot enables rapid visual prioritization of modules with strong relevance to OUD risk or to genotype- and treatment-dependent transcriptional responses. **(c) Top shared pathway enrichment across regionally prioritized modules.** Heatmap displaying the top 60 Gene Ontology Biological Process (GO:BP) pathways most frequently enriched across region–module–group combinations. Each column represents a region-specific module within a given experimental group. Each row is a GO:BP term, hierarchically clustered by pathway similarity. The fill color encodes the negative log_10_ of the Benjamini–Hochberg–adjusted P-value for pathway enrichment (see color bar), with higher values reflecting stronger enrichment. Region-specific modules were first identified using Module Degree Centrality (MDC) analysis, in which module eigengene expression was averaged within Allen Brain Atlas level-7 regions and modules were flagged as region-specific based on a z-score threshold (z > 1.5). For each region–module–group combination, pathway enrichment was performed using *enrichGO* (clusterProfiler, org.Mm.eg.db, “Biological Process” ontology), with the module gene set tested against the group-specific WGCNA background. The top 60 most frequently enriched pathways (across all combinations) were selected for visualization. Rows were hierarchically clustered by GO slim ancestors' categories. **(d,e,j,k) Atlas-projected differential co-expression networks of top modules in AA and GG backgrounds.** Representative brain-wide differential gene co-expression networks for the top green **(d, j)** and grey **(e, k)** WGCNA modules, selected based on their dynamic regulation in AA and GG backgrounds, respectively. Networks are visualized as overlays on the Allen Mouse Brain Atlas (sagittal slice, z=510), with nodes positioned at region centroids. For each genotype (AA, GG), group-specific inter-regional co-expression networks were constructed using module-level expression across major brain regions (CTX, CNU, IB, MB, HB). Nodes correspond to Allen CCFv3 level-7 regions and are colored by the top-enriched GO Biological Process pathway for that region and module (from SAL group; grey indicates no region-specific significant enrichment). Node labels indicate region acronyms. Edges represent inter-regional co-expression (Pearson correlation) within the module, with **edge color** reflecting the normalized change in connectivity between MOR and SAL conditions (Δcorrelation; red = gain of connectivity, blue = lost of connectivity), and **edge width** indicating baseline SAL network strength. Only the top 200 strongest edges per network are shown. This approach visualizes dynamic, region-resolved rewiring of module-specific gene co-expression in response to morphine exposure, highlighting both spatial and pathway-specific reorganization in opioid dependence. Green and grey modules were selected as representative dynamic modules for AA and GG, respectively, based on their ranking in differential network analysis (see Methods). **(f–i) Hub gene–centered subnetworks within representative dynamic modules across genotypes and treatments.** Subnetwork visualizations highlighting the top Δhub genes (highest absolute change in network centrality) and their immediate neighbors within the green and grey WGCNA modules for AA and GG backgrounds, corresponding to the differential network plots (see panels d/e/j/k). Panels show: (**f:** AA, green module | **g:** GG, green module | **h:** AA, grey module | **i:** GG, grey module). For each genotype and module, module gene expression was aggregated across matched brain regions in MOR and SAL conditions. Mutual information networks were inferred separately for each condition using the ARACNe algorithm (minet R package). Hub scores were defined as the number of direct connections per gene (network degree). The top three genes with the largest absolute change in hub score between SAL and MOR (Δhub score) were selected as Δhub genes. For each, the immediate neighborhood was extracted from the MOR-condition network, yielding a subnetwork comprising the Δhub genes (gold nodes) and their primary neighbors (sky blue). Edge width reflects the strength of gene–gene association. These plots highlight key genes whose network centrality is most strongly altered by morphine exposure within each module and genotype, revealing candidate regulators of opioid-induced network reorganization. Hub genes are annotated by name; neighbors are shown for contextual structure.

To systematically unravel how chronic opioid exposure reshapes gene expression across brain regions and genotypes, we first mapped region-specific morphine-responsive genes using inter-regional differential expression analysis (Fig. 4A), followed by comprehensive gene coexpression network (WGCNA) and pathway enrichment analyses to reveal both shared and genotype-specific programs of adaptation (Fig. 4B–K). Examination of region-specific morphine-responsive genes revealed both shared and divergent patterns between AA and GG genotypes (Fig 4A). For instance, in AA mice, *Pvalb* and *Spp1* were selectively upregulated in the VTA, whereas in GG mice, *Slc6a3* and *Cck* were VTA-specific DEGs. Some genes, such as *Ndrg4*, were morphine-responsive across multiple regions and genotypes, reflecting conserved neuroadaptive responses. However, the majority of DEGs exhibited striking region- and genotype-specificity, with GG brains showing greater activation of immune and cell stress pathways—such as upregulation of *Agt* and *Fth1* in VTA, thalamic and cortical regions—while AA brains favored genes related to synaptic plasticity and glial support, including *Snap25*, *Atp1b1*, and *Plp1*, especially in the prefrontal cortex and midbrain. These differences in the spatial patterning and composition of morphine-responsive genes set the stage for the distinct coexpression network architectures and hub gene dynamics observed in subsequent analyses (see full list of DEG in **Supplemental Table 1**).

We next applied consensus WGCNA across all groups to define ten core gene coexpression modules, each capturing a major axis of transcriptional covariation across the brain (see Methods; Fig. 4B). Module–trait correlation analysis revealed that each gene coexpression module is enriched in specific brain regions and exhibits distinct anatomical-dependent expression patterns (See Methods and **Supplemental Table 1**).

Each consensus WGCNA module mapped onto a distinct biological program and anatomical distribution. The green module encodes oligodendrocyte differentiation and myelin formation, predominantly activated in AA brains. The gray module highlights immune and neuropeptide signaling, with marked upregulation in GG brains under morphine. The blue module is enriched for genes involved in synaptic transmission and neuronal plasticity, while the brown module captures cortical neuron and GABAergic signaling programs. Pink and magenta modules are associated with astrocytic and hypothalamic networks, respectively, and turquoise and yellow modules reflect metabolic, mitochondrial, and peptide processing pathways. This modular framework provides the foundation for the detailed spatial and genotype-specific network adaptations described in subsequent analyses (Fig. 4B–K, Supplemental Fig. 7).

Given the substantial transcriptional overlap between brain regions, we performed shared pathway enrichment analysis (GO Slim) across the morphine-sensitive regions and group-level WGCNA modules to identify convergent core biological processes (Fig. 4C). We found that while morphine exposure altered the activity of many genes across the brain, the top shared pathways were region-and genotype-specific: the AA-morphine brain favored synaptic transmission and dendritic plasticity (particularly in prefrontal cortex), whereas the GG-morphine brain preferentially activated autophagy and cell-stress signaling (notably in the paraventricular thalamus). These pathway enrichments suggest that *Oprm1* risk alleles skew the brain’s response to chronic opioids away from healthy plasticity and towards maladaptive immune and cell-stress mechanisms.

To investigate how these transcriptional changes alter large-scale molecular wiring, we performed differential co-expression network (DE network) analysis, which reveals how module-specific gene connectivity patterns are dynamically rewired across brain regions by morphine and *Oprm1* genotype (Fig. 4D, E, J, K). We focused further network analysis on modules that showed the greatest genotype-mediated morphine response. For example, the green module reflects a striking, previously unrecognized mechanism of resilience: widespread transcriptional coordination of oligodendrocyte-related pathways specific to the protective AA genotype. Under morphine, AA brains show coherent upregulation of genes involved in axonal ensheathment, myelin membrane formation, and glial support across prefrontal, thalamic, and midbrain regions (Fig. 4D). The differential coexpression network for the green module (Fig. 4D, J) further emphasized genotype-specific reorganization. In AA mice, morphine induced new coexpression links especially along a FRP–thalamus–VTA–pons axis. This implies that AA mice under morphine develop tighter coordination between the frontal cortex, midline thalamus, midbrain VTA, and pontine nuclei within the green-module genes – potentially reflecting enhanced myelination or glial support along reward pathways. In GG, the strongest new connections skewed toward a septal (LS)–thalamus–superior colliculus axis. This suggests that GG morphine brains might reroute this network toward sensory/motor integration regions (colliculi) rather than the frontal–midbrain circuits.

In AA, the top Δhub genes (genes whose network centrality most increased with morphine) were *Plp1*, *Tcf12*, and *Rab31* — key regulators of oligodendrocyte differentiation and vesicular trafficking — and suggest a brain-wide mobilization of glial adaptation (Fig. 4F). These AA hub genes formed an interconnected subnetwork: *Plp1* connected with *Mag* and *Pllp* (myelin-associated glycoprotein and plasmolipin, both myelin components), while *Rab31* connected with vesicle and glial genes.This highly cohesive oligodendrocyte-centric hub suggests that in AA mice, morphine prompts a coordinated upregulation of myelination and glial support genes across the brain. The GG’s green-module key genes were *S100a16, Gab1*, and *Fth1*, which notably did not interconnect with each other (Fig. 4G). *S100a16* is a stress-responsive calcium-binding protein (in glia), *Gab1* an adapter in growth factor signaling, and *Fth1*, the ferritin heavy chain gene, is important in iron storage. Their top neighbors included a mix of oligodendrocyte genes (*Fa2h* and *Ugt8a*, two genes encoding enzymes required for myelin lipid synthesis, also found in AA networks) and inflammatory or stress markers (*Spp1*/Osteopontin, an activated microglia/astrocyte cytokine; *Sall1*, a microglial identity factor). The failure of the GG brain to activate a coherent glial repair program may render it more vulnerable to the synaptic destabilization caused by chronic opioids.

The gray module is the strongest transcriptional signature of addiction susceptibility uncovered in this study. Critically, we observe selective enrichment of “cell killing” pathways within the nucleus accumbens and substantia innominata in GG, but not AA mice. The differential coexpression networks for gray show that morphine drives widespread network “gain of connectivity” in both genotypes, but with notable qualitative differences. In GG brains, this network shows widespread morphine-induced activation across thalamic, striatal, and cortical regions (Fig. 4E, 4K).

Hub genes in the gray module highlight an immune theme. In GG, top hub genes were *Mc5r, Sox2, and Cd55* which are highly interconnected with many immune and neuropeptide genes. For instance, Sox2–Avp–Gba–Ntrk1–Cd55 formed a chain linking a neuroendocrine hormone (*Avp*, arginine vasopressin) and a lysosomal enzyme (*Gba*, glucocerebrosidase) to the hub nodes. Another chain, Sox2–Tyrobp–(Cd55), connected via *Tyrobp* (DAP12), a microglial adapter that drives phagocytosis and inflammation. Moreover, *Ifng* (interferon-γ), *Oxt* (oxytocin), *Gal* (galanin), *Pde10a* (phosphodiesterase in striatal neurons) were neighbors to Sox2 or Cd55. This mix of pro-inflammatory cytokine (*Ifng*) and neuropeptides (AVP, OXT, galanin) suggests crosstalk between immune and neuroendocrine stress systems in morphine-exposed GG mice. In contrast, in AA mice, the top morphine-affected hubs were *Cd55, Mid1, and H2-Ab1. Cd55* and *Mid1* shared neighbors like *Ntrk1* (TrkA neurotrophin receptor) and *Slc5a7* (choline transporter), suggesting links to neuronal survival/growth factors. *H2-Ab1*stands apart with only *Cd74* as a neighbor, consistent with an antigen-presentation subcomponent. Thus in AA, gray-module hubs indicate a modest immune activation: upregulation of complement regulators and antigen presentation, perhaps reflecting microglia responding to opioids in a controlled way (expressing MHC-II and complement inhibitors, possibly to clear debris while preventing excessive damage).

While we focused our analysis on the Green and Gray modules due to the striking difference in gene co-expression network dynamics, we also identified several other modules of interest (Supplemental Fig. 7). For example, in the Pink module, morphine exposure drives genotype-dependent network shifts of astrocyte-specific genes. In AA mice, hubs such as *Gpr37l1*, *Lpin1*, and *Daam1* form cohesive subnetworks supporting astrocytic energy homeostasis, cytoskeletal remodeling, and neurotransmitter clearance. These likely contribute to a neuroprotective astrocyte phenotype. In contrast, GG mice show a reactive astrocyte profile with activation of *Apoe*, *Glul*, and *Cd81*. These hubs interact with stress response genes (*Vim*, *Rgs5*) indicative of an injury-response phenotype. In the Blue module (synaptic plasticity) both genotypes upregulate synaptic vesicle and receptor trafficking genes following morphine exposure. AA-specific hubs (*Camk2n1*, *Nckap1*) point to cytoskeletal regulation and CaMKII inhibition, suggesting neuroadaptive refinement. GG-specific hubs (*Gnao1*, *Cyfip2*) shift toward translational control, highlighting altered synaptic efficiency. Lastly, in the Magenta and Turquoise modules, GG-specific mitochondrial stress, autophagy, and neuropeptide dysregulation are prominent in the PVT and hypothalamus. Notably, *Nap1l5* and *Pcsk1n*—poorly characterized in addiction—emerge as hubs linking genotype to peptide-processing control. Together, these modules define how the brain organizes opioid adaptation into circuit-specific compartments, emphasizing that the A118G variant reshapes not only which genes are activated or repressed, but also how and where coexpression is structured.

## Discussion

Using spatial transcriptomics at cellular resolution, we identified the molecular and cellular adaptations in the opioid-dependent mouse brain in the context of genetic risk conferred by the *Oprm1* A112G SNP. By profiling cell type-specific transcriptional changes in *Oprm1*AA (protective allele for OUD) and *Oprm*1GG (risk allele for OUD) mice, we uncovered unexpected shifts in cell type distributions and molecular responses across brain regions following chronic opioid exposure.

A key discovery from our study is that opioid-induced changes in cell states varied markedly by genotype. While both AA and GG mice exhibited regionally specific alterations in cell states following drug exposure, AA mice displayed a more pronounced shift in glutamatergic neuronal populations, with broad-scale enrichment of glutamatergic cell groups across multiple brain structures in the opioid-dependent state. This genotype-dependent enrichment was particularly evident in cortical and subcortical areas, suggesting that glutamatergic plasticity in AA mice may contribute to differences in opioid response. In contrast, GG mice exhibited a more constrained reorganization of neuronal populations, but notable enrichment of specific oligodendrocyte clusters. We hypothesize that the increased abundance of oligodendrocytes in the pons and medulla of GG mice following opioid exposure may reflect a neuroadaptive response to prolonged opioid use, potentially linked to myelination and neural repair mechanisms. Given that opioids modulate neuronal excitability and induce plastic changes in the mesocorticolimbic system, this differential adaptation in neuronal versus glial populations may contribute to the distinct network-level instability previously reported in GG mice^20^.

Another unexpected finding was that, while opioid-dependent transcriptional changes in neurons were largely confined to glutamatergic and GABAergic populations in select brain regions, glial cells—including astrocytes, microglia, and oligodendrocytes—exhibited widespread and pronounced transcriptional alterations across the entire brain. scRNA sequencing in the nucleus accumbens of mice identified gene expression changes unique to oligodendrocytes following acute morphine^14^. A human postmortem study in ventral midbrain found broad alterations in transcriptomes of microglia, oligodendrocytes and astrocytes^13^. While these individuals all had a history of opioid misuse, no genetic information was available. In our study, we observed that oligodendrocytes underwent substantial transcriptional shifts in GG mice, with disproportionate activation of genes involved in neuroinflammation (*Atrn*, *Ip6k2*), immune signaling (*Tgfbr1*, *Cd14*, *Selplg*), and synaptic regulation (*Kcnq5*). A second postmortem study of individuals with OUD found enrichment of genes that control proinflammatory cytokines in Dorsolateral prefrontal cortex and nucleus accumbens^19^. Together with our data these findings suggest that non-neuronal cells play a more extensive role in opioid-induced neuroplasticity than previously recognized. These changes may reflect an increased susceptibility to opioid-induced neuroinflammation in GG mice, further supporting a genotype-dependent distinction in opioid response.

A key question that arises from these findings is whether glial-specific transcriptomic changes are solely a consequence of opioid exposure or whether they also reflect underlying genotype-dependent differences. While both AA and GG mice exhibited drug-induced glial adaptations, the specific genes and pathways involved varied by genotype, suggesting that inherited risk influences the cellular and molecular response to chronic opioid use. This distinction highlights the role of *Oprm1* in shaping not only neuronal responses to opioids but also broader neuroimmune and glial plasticity.

To our knowledge, this is the first cellular-resolution spatial transcriptomics study that integrates genetic risk with the effects of chronic opioid exposure. These data provide a valuable resource for the field of opioid addiction and offer novel insights into the molecular mechanisms by which a single nucleotide polymorphism in *Oprm1*, a key determinant of addiction vulnerability in both mice and humans, reshapes the transcriptional and cellular landscape of the brain. By mapping gene coexpression, pathway activity, and network remodeling across cell types and brain regions, our study reveals how *Oprm1*-driven genetic risk is translated into discrete, anatomically organized changes in glial, neuronal, and network function—defining the molecular logic by which opioid dependence emerges from individual genetic background.

## Methods

### Animals

Adult female mice homozygous for the *Oprm1* A112G knock-in allele (Oprm1^A112G^/Oprm1^tmJabl^), which corresponds to the human *OPRM1* A118G single nucleotide polymorphism (SNP), were used in this study. This model has been extensively characterized in previous studies^8,9,37–41^ to assess its impact on opioid-related behaviors and receptor function. Mice were housed under standard laboratory conditions with a 12-hour light/dark cycle and provided *ad libitum* access to food and water. All experiments were performed in accordance with institutional animal care and use guidelines (see Ethics Approval).

To model opioid dependence, female mice with *Oprm1* AA or GG alleles were randomly assigned to chronic morphine (escalating doses of morphine sulfate (20, 40, 60, 80, and 100 mg/kg, i.p., twice daily) or saline groups (n = 3/ genotype/ treatment; total n = 12). This sample size aligns with established standards for high-resolution spatial transcriptomics studies, where tissue-wide sampling across hundreds of fields of view per animal provides robust statistical power even with relatively small animal cohorts^42,43^.

The morphine group received escalating doses of morphine sulfate (NIDA Drug Supply Program 20, 40, 60, 80, and 100 mg/kg, i.p., twice daily) or saline over the same 5-day period to induce a morphine-dependent state. This escalating morphine dosing regimen is widely established in rodent models to reliably induce opioid dependence while minimizing acute toxicity, enabling robust study of chronic adaptation mechanisms^44,45^.

### Tissue Preparation

Thirty minutes following the final injection (saline or morphine), mice were deeply anesthetized with sodium pentobarbital (50 mg/kg, i.p.) and transcardially perfused with 50 mL of ice-cold phosphate-buffered saline (PBS, pH 7.4), followed by 50 mL of ice-cold 4% paraformaldehyde (PFA, pH 7.4). Brains were post-fixed in 4% PFA at 4°C for 48 hours, then washed three times for 30 minutes each in cold PBS before transferring to 70% ethanol for dehydration. Brains were paraffin-embedded at 60°C and stored at 4°C until sectioning.

### Spatial Transcriptomics – Sectioning

Paraffin-embedded brains were sagittal sectioned at 5 μm thickness using a rotary microtome. For each animal, one midline sagittal section corresponding to lateral coordinates 0.475–0.674 mm (Allen Mouse Brain Atlas CCFv3; 10 μm resolution) was selected for analysis. Sections were mounted on Leica BOND PLUS slides (Leica Biosystems, Cat. no. S21.2113.A, by the Molecular Pathology and Imaging Core at the University of Pennsylvania. All sectioning and mounting were performed under blinded conditions, and consistent anterior-posterior orientation was maintained across samples. No cryoprotection was performed due to paraffin-based tissue processing.

### In Situ Hybridization and CosMx Spatial Molecular Imaging

Tissue processing and spatial transcriptomic imaging were performed using the CosMx^™^ Spatial Molecular Imager (NanoString Technologies) according to manufacturer protocols for FFPE samples (Slide Preparation Manual MAN-10159–01; Instrument Manual MAN-10161–01). Sagittal brain sections (5 μm) were cut from formalin-fixed, paraffin-embedded tissue blocks and mounted on Leica BOND PLUS slides (Cat. no. S21.2113.A). Slides were baked overnight at 60 °C, deparaffinized in xylene (2 × 5 min) followed by 100% ethanol (2 × 2 min), and subjected to heat-induced target retrieval using 1× NanoString Retrieval Buffer for 15 min at 100 °C in a pressure cooker. Sections were washed in ethanol and DEPC-treated water, then permeabilized in Proteinase K solution (3 μg/mL) for 40 min at room temperature. Tissue was post-fixed in 4% paraformaldehyde (PFA) for 20 min and hybridized with fiducial oligonucleotides for 20 min before crosslinking with 0.1 mg/mL sulfo-NHS-acetate for 25 min. Probe hybridization was carried out overnight (~16 h) at 40 °C in a humidity-controlled chamber.

Each slide was hybridized with the CosMx Mouse Neuroscience RNA Panel (1,000-plex; 950 genes and 10 internal controls), supplemented with a custom-designed 50-plex panel to expand detection of glial subtype markers, ligand–receptor pairs, and transcripts spanning 72 functional pathways (see Supplementary Table 1 for complete gene list and annotations). Negative control probes provided by NanoString (listed in Supplementary Table 1) were included in each hybridization reaction to assess transcript detection accuracy, specificity, and sensitivity. Negative controls facilitate background correction and noise estimation.

Post-hybridization, slides underwent stringency washes and multiplexed segmentation staining with DAPI, rRNA, histone H3, and GFAP, followed by signal stabilization, flow cell assembly, and system readiness verification prior to imaging.

### Imaging and Field-of-View (FOV) Acquisition

Slides were imaged using the CosMx^™^ Spatial Molecular Imager (NanoString Technologies), a high-throughput, automated benchtop fluorescence imaging platform configured for subcellular transcript detection. The instrument supports fully automated imaging and fluidics, with up to four slides processed in parallel per run. After slide and flow cell loading, the system performs automated tissue scanning and tissue detection to assist in defining the optimal imaging area. Fields of view (FOVs) were selected using the CosMx SMI Control Center interface to maximize anatomical coverage across major brain divisions, including the cerebral cortex, cerebral nuclei, interbrain, midbrain, and hindbrain. Each FOV measured 0.5 × 0.5 mm, and an average of 393 FOVs were collected per sample in this study to ensure adequate spatial representation.

### Signal detection and cyclic reporter hybridization

Signal detection was achieved via cyclic fluorescent probe hybridization using the CosMx^™^ Spatial Molecular Imager (NanoString Technologies). The detection chemistry is non-enzymatic and relies on hybridization of fluorescently labeled reporter oligonucleotides to target-specific decoder probes. During each imaging round, a defined subset of reporter probes was hybridized to the sample. Following image acquisition, the reporters were removed using a low-salt strip wash buffer, and a new reporter set was applied in the next cycle. This process was repeated over multiple rounds, with the total number of hybridization–imaging–stripping cycles determined by panel complexity (e.g., 32 reporter cycles for 1,000-plex panels in this study).

Four proprietary imaging buffers were used during each run: a strip wash buffer to remove hybridized reporters, a reporter wash buffer to clear unbound reporters, an imaging solution buffer to optimize fluorescent signal detection, and an immersion solution buffer to maintain stable water immersion between the objective and flow cell coverslip.

Imaging was performed on the CosMx SMI platform using a high-resolution inverted fluorescence microscope (22.77× water-immersion objective, multichannel filters, temperature-controlled hybridization chamber) across four fluorescent channels (488/512 nm, 530/553 nm, 590/630 nm, and 656/684 nm), enabling cyclic subcellular transcript detection at ~200 nm resolution.

### Image Processing and Transcript Quantification

Image acquisition, stitching, background correction, and transcript quantification were performed using CosMx SMI Control Software and Data Analysis Suite v1.2. High-resolution imaging across multiple hybridization rounds was spatially registered using fiducial markers, and fields of view were stitched to reconstruct whole-slide architecture. Background subtraction was guided by negative control probes, and single-molecule spot detection was performed using intensity thresholds and point spread function modeling. Detected transcripts were assigned to segmented cells using morphological markers (DAPI, rRNA, histone H3, GFAP) to generate per-cell gene expression matrices. Spatial coordinates were preserved for downstream analyses including density mapping, neighborhood computation, and region-level quantification, all processed within the AtoMx Spatial Informatics Platform.

### Quality Control Metrics and Run Performance

Run metrics were evaluated for each slide, including the number of cells detected, average transcript count per cell, 9th percentile transcript coverage, and mean per-cell negative probe counts (see Supplementary Table 1 for full QC metrics). Across all four slides, the number of detected cells ranged from 251,629 to 290,746, with mean transcript counts per cell ranging from 367 to 697. The 9th percentile transcript counts per cell ranged from 835 to 1,481 and mean negative control probe counts per cell remained below 0.54 on all slides. All slides passed internal manufacturer-defined quality control thresholds (NanoString Technologies, Data Analysis Manual MAN-10162–03). Outlier test over Negative Probe showed 100% pass rate. Fields of view were excluded from downstream analyses if they exhibited any of the following: average transcript counts below 100 per cell, more than 1.0 negative control probe count per cell, or fewer than 500 detected cells per FOV. These quality control thresholds—including the requirement of at least 500 cells per field-of-view (FOV) and average transcript counts ≥100 per cell—were set according to NanoString's established guidelines for CosMx^™^ Spatial Molecular Imaging, resulted in minimal exclusion, with 2% of FOVs removed, confirming the overall high quality and uniformity of spatial transcriptomic data.

### Data Analysis

#### Gene Expression Quantification

Raw spatial transcriptomic data were transferred directly from the CosMx^™^ Spatial Molecular Imager to the AtoMx^™^ Spatial Informatics Platform (NanoString Technologies) for preprocessing and quality control using the CosMx RNA QC pipeline (Data Analysis Manual v1.2). Cell- and probe-level quality control was performed to remove low-quality observations. Negative control probes were screened using Grubb’s test (P < 0.01), and flagged probes were excluded from downstream normalization. Cells were retained if they contained ≥5 total transcripts, had ≤10% of counts assigned to negative control probes, exhibited a total count-to-detected gene ratio >1, and did not exceed outlier thresholds for segmentation area (P < 0.01). These initial QC thresholds were intentionally permissive, as this preprocessing step served primarily to remove gross artifacts and retain the majority of cells for subsequent spatial registration. A more stringent quality control filter was later applied prior to cell-type annotation and clustering (see Clustering and Cell-type Identification), ensuring that downstream analyses were based on high-confidence transcriptomic profiles. Fields of view (FOVs) with fewer than 10 transcripts per cell on average were excluded. Targets with expression below the 50th percentile of the negative control distribution or with background-corrected detection P-values >0.01 were also excluded.

Across all samples, a total of 1,053,656 cells were detected. After filtering, 949,137 high-confidence cells were retained for downstream analysis (pass rate: 90.1%). Normalized expression values were computed using the *LogNormalize()* function in the Seurat v4.3 package in R^46^. For each cell, transcript counts were scaled to 10,000 total UMIs and log-transformed. Following normalization, no significant batch effects were observed across flow cells or slides. Batch effects across slides and flow cells were formally assessed using principal component analysis (PCA) and visual inspection of PCA plots, ensuring that no obvious clustering correlated with experimental batches or technical replicates was present. Gene expression matrices and cell-level metadata were exported from AtoMx in tab-delimited format and imported into Seurat for all subsequent analyses, including clustering, visualization, and differential expression.

#### Spatial Registration and Anatomical Annotation

Following quality control and normalization, single-cell expression data exported from Seurat were processed using a custom multimodal computational pipeline, **CellDynamicST**, developed in the lab for high-precision anatomical mapping. This workflow performs geometric correction, orientation normalization, and field-of-view (FOV) reconstruction by aligning raw (x, y) cell coordinates into a unified tissue coordinate system (Supplemental Fig. 1). To register each brain section to a common anatomical framework, we implemented **STSHARCQ** (Spatial Transcriptomic Slice Histology Alignment, Registration, and Cell Quantification), a tool adapted from SHARCQ for atlas-based alignment of histological sections^47^. STSHARCQ performs iterative slice-to-atlas registration, correcting for in-plane rotation, brain tilt, and nonlinear tissue deformation. Each cell was mapped to a brain atlas coordinate space using the Allen Mouse Brain Common Coordinate Framework (CCFv3), and anatomical identities were assigned through hierarchical back-tracing of parent regions within the ontology tree. FOVs from each slide were registered with their corresponding anatomical location and experimental condition. Final cell-level annotations included the associated Allen Atlas structure at each hierarchy level (e.g., structure ID, acronym, and structure depth), enabling spatially resolved comparisons across conditions. Regions were considered matched based on identical anatomical identification within the Allen CCFv3 level-7 anatomical hierarchy, ensuring rigorous spatial comparability in differential expression analysis consistent with established spatial transcriptomics standards^48^. The STSHARCQ registration tool, bundled within our CellDynamicST platform, will be publicly released with full documentation and example datasets to ensure methodological transparency and reproducibility.

#### Clustering and Cell-type Identification

Cell-type clustering was performed independently for each genotype and treatment condition using a generalized clustering workflow implemented in the **CellDynamicST** platform. This pipeline combines unsupervised reference-based clustering with supervised label propagation to capture treatment- and genotype-specific variation while maintaining cell-type consistency. Prior to clustering, we applied quality control filters to exclude low-quality cells. Specifically, we removed cells with total transcript counts (*nCount_RNA* > 181) or detected gene counts (*nFeature_RNA* > 99) below the 5th percentile, eliminating cells with poor capture efficiency or low sequencing depth.

A subset of cells from saline-treated Oprm1 AA brains (opioid-naïve baseline) was designated as the reference set. We selected cells from saline-treated Oprm1 AA mice as the baseline reference set due to their genetically wild-type opioid receptor status and absence of morphine exposure. Pooling samples from treatment or genotype variants during initial clustering can bias cluster definitions toward condition-specific transcriptional profiles, confounding subsequent cell-type annotations across conditions^49,50^. Thus, using a clearly defined baseline condition facilitates robust, unbiased annotation and accurate cross-condition mapping. These cells were normalized and scaled using standard preprocessing in Seurat v4.3^46^. Principal component analysis (PCA) was conducted on the top 100 components. To remove technical bias, components with Pearson correlation >0.7 to a technical bias vector—defined as log_2_-transformed total gene counts per cell—were excluded^51^.

Neighborhood graph construction was performed on the reference subset using Seurat’s *FindNeighbors()* function with the Annoy algorithm (search trees = 100, k = 50, using 59 PCA dimensions). Louvain clustering with multilevel refinement was applied to identify cell clusters (*FindClusters()* function, resolution = auto). The identified reference clusters were assigned putative cell-type identities based on marker gene expression and were used as training labels for a random forest classifier (number of trees = 200). This supervised model was then used to predict cell types in the remaining experimental cells (i.e., from morphine-treated and/or Oprm1 GG mice). Predicted cell identities were appended to the full expression matrix and used in subsequent generalized PCA, neighborhood graph analysis, and visualization. To visualize the global cell landscape, we downsampled up to 1,000 cells per cluster and computed two-dimensional projections using t-distributed stochastic neighbor embedding (t-SNE) on corrected PCA components^51^. This hybrid clustering strategy enabled robust detection of genotype- and treatment-dependent shifts in cellular states while maintaining coherence across datasets.

#### Neurotransmitter and Glial Cell-type Classification

To assign neurotransmitter identity, canonical transporter and biosynthesis gene pairs were evaluated using a probabilistic classification framework. For each neurotransmitter-related gene, log₂(counts per million) was computed, and a two-component Gaussian mixture model (GMM) was fit to distinguish low- vs. high-expression modes. A consensus threshold of log₂CPM > 3 was applied to define high-expressing cells. Cells were assigned to a neurotransmitter-type if they met established criteria:

- **Glutamatergic**: Slc17a6, Slc17a7, or Slc17a8
- **GABAergic**: Slc32a1 or Slc18a2, plus Gad1, Gad2, or Aldh1a1
- **Glycinergic**: Slc6a5
- **Cholinergic**: Slc18a3 and Chat
- **Dopaminergic**: Slc6a3 or Slc18a2, plus Th and Ddc
- **Serotonergic**: Slc6a4 or Slc18a2, plus Tph2 and Ddc
- **Noradrenergic**: Slc6a2 or Slc18a2, plus Dbh
- **Histaminergic**: Slc18a2 and Hdc

Clusters were assigned a neurotransmitter identity if >30% of their constituent cells met the threshold-based criteria^51^.

Non-neuronal clusters were similarly annotated using a GMM-based approach applied to curated glial marker modules. Each glial subtype required high expression in at least one gene from each of two biologically distinct modules:

- **Astrocytes**: structural (Gfap, Aldoc, S100b) and functional (Slc1a3, Aqp4, Agt)
- **Microglia**: surface receptors (Cx3cr1, Csf1r, Itgam) and immune effectors (Iba1, C1qc, Tmem119, P2ry12)
- **Oligodendrocytes**: myelin-associated (Plp1, Mbp, Mobp, Mag) and transcriptional (Olig1, Olig2, Sox10, Nkx2-2)
- **Ependymal**: ciliary (Foxj1) and secretory (Ttr, Aqp1)
- **Endothelial**: tight junction (Cldn5, Pecam1, Vwf) and vascular receptor (Flt1, Kdr)

Clusters were annotated as glial subtypes if >30% of cells passed thresholds in both modules. This cell-type classification threshold (>30% of cells within a cluster) was adopted following precedent from spatial transcriptomic annotations established by Yao et al., 2023, demonstrating robust classification performance and biological interpretability at this threshold.

#### Hierarchical Cell-type Annotation

Final cell-type annotation was constructed hierarchically using: (1) inferred neurotransmitter or glial identity, (2) cluster-level marker gene profiles, and (3) anatomical localization. Clusters sharing neurotransmitter/glial identity and anatomical distribution, but differing in marker genes, were grouped into mesostructural subtypes. These were further aggregated into neuronal or non-neuronal superclasses. Clusters not meeting classification criteria were labeled “Other.” This resulted in a four-level hierarchy: cluster identity → mesostructural subtype → subtype → neuronal/non-neuronal category (Fig. 1A, Supplemental Fig. 2).

#### Validation and Refinement of Spatial Cell-type Annotations

Although initial cell-type annotations were derived from molecular markers and structural enrichment patterns, we observed that spatial transcriptomic annotation alone could result in partially ambiguous or mixed structural assignments—particularly at coarse anatomical levels (e.g., Allen CCFv3 depth 3 meso-structures). In some cases, rare combinations of neurotransmitter identity and anatomical localization emerged, warranting further manual review.

To address these limitations, a secondary refinement step was implemented. Cell distribution maps were generated for each annotated cluster based on spatial density across individual brain samples. These maps included the inferred meso-structure identity (from Allen CCFv3), dominant neurotransmitter category, and estimated cell-type proportions. A blinded experimenter, unaware of genotype or treatment condition, manually reviewed each cluster using the spatial and metadata overlays. When appropriate, structural annotations were refined to deeper atlas levels (depth > 3) to improve anatomical specificity. Clusters with inconsistent or uncertain localization were flagged for further evaluation. Manual refinement prioritized anatomical annotations between Allen CCFv3 hierarchical levels 3 through 7. Levels 1–2 provide insufficient granularity (e.g., simple distinctions between cerebrum and brainstem), while from level 3 onward, structural definitions (e.g., cortex [CTX], cerebral nuclei [CNU], interbrain [IB], midbrain [MB]) allow meaningful regional specification relevant to opioid dependence^20^. This refinement strategy ensures biologically precise anatomical localization.

Following manual inspection, all clusters were subjected to an additional quality control step to assess transcriptional distinctiveness. Marker gene analysis was performed using Seurat (*FindMarkers()*), and clusters were retained only if they met the following criteria: at least 8 high-confidence markers (adjusted P < 0.01), relative enrichment (q_diff) ≥ 0.7, and a cumulative differential expression score (DE score) ≥ 150, computed from the sum of gene-level −log_10_(P_adj) values. Clusters failing to meet these criteria were excluded from downstream analyses. The thresholds for marker gene selection were empirically optimized based on sensitivity analyses to balance detection sensitivity with specificity, ensuring stable and biologically informative clusters. This approach aligns with common practices in single-cell and spatial transcriptomics analyses, where thresholds are often tailored to the specific dataset and analytical goals to effectively distinguish meaningful cell populations^43^.

To assess the biological validity of annotated cell types, we further cross-referenced results with the CellMarker2 mouse brain database^52^. Only markers corresponding to brain tissue were retained. For each cluster, a filtered set of high-confidence markers (log_2_FC > 1, min.pct ≥ 0.4, q_diff ≥ 0.7, adjusted P < 0.01) was used to compute an overlap score, defined as the proportion of cluster-specific markers intersecting with database-defined gene sets. For each cluster, the best-matching CellMarker2 cell type was identified based on maximal overlap. A bar plot of overlap scores was generated and saved for all clusters, alongside summaries of best-match annotations, overlap scores, and marker gene composition (Supplementary Figure 3 & **Supplementary Table 1)**. While canonical neuronal and glial identities showed strong agreement with CellMarker2, some clusters exhibited no close match—highlighting the broader annotation capacity of our spatial transcriptomics pipeline, particularly for region-specific or transcriptionally novel populations.

#### Differential Expression and Functional Enrichment Analysis

Differential gene expression (DGE) analysis was performed for clusters containing ≥50 cells using the *FindMarkers()* function in Seurat (v4.3), with minimum expression detected in 25% of cells (min.pct = 0.25) and a log_2_ fold-change threshold of 0.1. Only upregulated genes were retained for cluster-level marker identification. For visualization, the top three and top twenty marker genes per cluster were reported (Supplementary Figure 2 & **Supplementary Table 1)**, and complete marker gene lists were deposited online.

To assess spatially resolved gene expression variation, we conducted inter-regional DGE analysis across Allen Brain Atlas CCFv3-defined level 5 anatomical structures. Within each genotype and treatment condition, 500 cells per region were randomly subsampled, and DGE testing was performed using a Wilcoxon test with Benjamini–Hochberg adjustment (adjusted P < 0.01, |log_2_FC| > 1.5). Out of 47 regions, eight were prioritized for focused comparison based on DEG burden and biological relevance: lateral septal complex (LSX), ventral tegmental area (VTA), ventral striatum (STRv), pallidum (PALv), periaqueductal gray (PAG), isocortex, thalamus (TH), and hypothalamus (HY). DEGs between regions, genotypes, and treatments were visualized using Venn diagrams and volcano plots, with treatment-by-region interactions identified (padj < 0.01, |log_2_FC| > 1.5).

#### Spatial and Regional Gene Ontology Enrichment

Functional annotation was conducted using a two-part gene ontology (GO) enrichment framework. First, cell-level GO enrichment scores were computed to reveal spatial patterns of functional susceptibility to opioid exposure. Differentially expressed genes (DEGs) from saline versus morphine comparisons were ranked and weighted by log_2_ fold-change within each genotype. GO term gene sets were obtained from *GO.db* and *org.Mm.eg.db*, and enrichment scores were calculated for each cell using a weighted gene set enrichment analysis (GSEA)-based approach. Enrichment was computed separately across Biological Process (BP), Cellular Component (CC), and Molecular Function (MF) categories.

For each cell, GO enrichment scores were summarized and used to annotate cells as “enriched” or “depleted” for specific GO terms based on percentile thresholds. Spatial projection plots were generated across sagittal sections to visualize functional enrichment at single-cell resolution. In these maps, orange indicates MOR-enriched cells, and blue indicates MOR-depleted cells, relative to saline treatment. Spatial enrichment maps and GO term summaries are presented in Supplementary Table 1 and Figures 3.

Second, region-level GO enrichment was performed using a custom pipeline to compare treatment- and genotype-dependent transcriptional programs across major brain divisions (cerebral cortex, cerebral nuclei, interbrain, midbrain, hindbrain). DEGs from comparisons between AA SAL vs. AA MOR and GG SAL vs. GG MOR were used as input to *enrichGO()* (clusterProfiler v4.0), with enrichment calculated separately for BP, CC, and MF categories. For each brain region, top enriched terms were ranked using a composite metric incorporating adjusted q-value and average log_2_ fold-change across DEGs.

Results were visualized as paired bar plots, with overlaying dot plots showing log₂ fold-change (dot size) and q-value (dot color) for each GO term within AA and GG samples. Differential susceptibility was highlighted using binary color overlays: orange indicates GG-biased enrichment, and blue indicates AA-biased enrichment, based on between-genotype differences in weighted enrichment scores. This visualization framework enabled side-by-side comparison of transcriptional vulnerability to chronic opioid exposure across both ontological and anatomical dimensions.

#### Cell-type Distribution Dynamics in Opioid Dependence

To assess cell-type compositional dynamics during opioid dependence across genetically distinct backgrounds, we quantified and visualized the redistribution of annotated cell types across fine-scale anatomical structures (Allen Mouse Brain Atlas, CCFv3, depth 7). Cells from each experimental group (AA SAL, AA MOR, GG SAL, GG MOR) were stratified by brain region and cell-type annotation. Regional cell-type counts were aggregated into a matrix, and only cell types and brain regions present in all four groups were retained to enable fair cross-condition comparisons.

Morphine-induced changes in spatial abundance were calculated as the percentage change in cell counts relative to saline-treated controls within each genotype. To standardize these values for comparison, we applied min–max normalization followed by log transformation. Baseline abundance in the control condition was visualized via circle size in the heatmap, enabling joint interpretation of both absolute and relative changes. Circle radii were robustly scaled using clipped log-transformed counts between the 5th and 95th percentiles, to avoid distortion from rare, high-abundance outliers and enhance perceptual clarity across conditions.

We designed circle-encoded dot heatmaps, where:

- **Circle color** reflects the direction and magnitude of morphine-induced change (blue = depletion; orange = enrichment),
- **Circle size** represents the relative baseline abundance of each cell type in each region under control conditions,
- **Row and column ordering** follows the anterior–posterior mean spatial location, preserving neuroanatomical topology.

To improve interpretability, multilevel annotations were incorporated. Top annotations indicated mesostructural identity (e.g., cortex, hypothalamus), while bottom annotations displayed the proportional composition of major cell-type categories within each region.

Right-side annotations captured per-cell-type profiles, including neurotransmitter type distributions, endogenous receptor class composition, and Shannon diversity index, which quantifies anatomical breadth. Complementary Gini coefficients were computed per region to assess intra-regional inequality in cell-type representation.

This allowed for clearer visualization of morphine-responsive populations while maintaining consistent structure across AA and GG plots. The full dataset, including all observed cell-type distributions without filtering or subsetting, is provided in Supplementary Figure 2. A minimum cell count threshold was enforced during aggregation (n ≥ 50 per group) to reduce noise from undersampled clusters.

Together, this analytic framework reveals genotype-specific reorganization of brain-wide cellular landscapes in response to chronic opioid exposure, providing a spatial map of morphine-induced plasticity at single-cell resolution.

#### Treatment-Associated Transcriptional Disproportionality Across Cell Types (Fig 1G,H and Supplemental Fig 4)

To identify cell populations disproportionately contributing to the transcriptional response to chronic opioid exposure, we adapted a recently established framework^53^ originally developed to profile sex- and estrous cycle–dependent gene expression. While the original method was designed for bulk and single-cell transcriptomic datasets, we modified its core principles to accommodate the distinctive signal structure and sparsity patterns of high-resolution spatial transcriptomics data.

Specifically, for each genotype (AA or GG), we used DEGs identified from intra-genotype treatment comparisons (AA MOR vs. AA SAL and GG MOR vs. GG SAL). For each cell type, we calculated the average normalized expression of these DEGs and the mean absolute log_2_ fold change (|log_2_FC|) derived from the group-level comparison. Cell types with nonzero expression for fewer than a defined fraction of DEGs were excluded to avoid artifacts from underpowered clusters.

To robustly detect disproportionate expression, we computed a composite expression score for each cell type by averaging across all valid DEGs, then z-scored these values across all annotated types. Cell types were retained if they exceeded a two-sided 90% confidence interval (α = 0.1; z ≈ ±1.64) and if their mean |log_2_FC| exceeded 1. This dual-threshold approach ensures both statistical and biological relevance—filtering out cell types with marginal expression differences or low DEG concordance.

Z-scored “disproportionality scores” were visualized using precomputed t-SNE projections, colored via a coolwarm midpoint-normalized colormap. Text annotations were overlaid using adaptive label placement to minimize crowding. This visualization highlights transcriptionally “dominant” populations—i.e., those cell types that not only express many DEGs, but do so at higher-than-expected levels given the background distribution. While our analytical design follows the foundational structure proposed in Knoedler et al., the application to spatial transcriptomic data necessitated key adjustments:

- **Sparse expression distributions**: Spatial datasets often include dropout effects and low-abundance transcripts, requiring preprocessing filters (e.g., minimum expression coverage) not used in the original pipeline.
- **Normalization and scaling**: We applied total-count normalization and log1p transformation, followed by z-scoring within each cell-type distribution to control for global expression heterogeneity.
- **Noise suppression**: To reduce false positives, we imposed an additional log_2_FC threshold and used absolute rather than signed fold changes to prioritize magnitude over directionality.

This analytical framework identifies transcriptionally hyperresponsive cell types—populations where treatment-related DEGs are both overrepresented and highly expressed. These populations are likely to serve as key effectors of brain-wide remodeling in the opioid-dependent state and may help pinpoint subtype-specific vulnerabilities.

While this method emphasizes gene-level expression rather than cell-type abundance, the two metrics are complementary: a cell type may remain numerically stable while showing profound molecular reorganization. Results from this analysis were integrated with prior distributional and enrichment findings to inform broader interpretations of genetic susceptibility and regional specificity in opioid dependence. This approach may under-detect rare or transcriptionally quiescent populations. However, we provide genotype-specific t-SNE plots that include all annotated clusters, including those that did not meet statistical thresholds—as Supplementary Fig. 4, ensuring transparency and completeness.

### Inter-regional Co-expression Network Construction and Module Analysis

#### Inter-regional Gene Co-expression Analysis (Supplemental Fig 6)

To examine how the Oprm1 A118G variant and chronic opioid exposure remodel transcriptional coordination across anatomically distinct brain regions, we constructed group-specific inter-regional co-expression networks from spatial transcriptomic data. This analysis aimed to identify long-range transcriptional coupling patterns that may underlie genotype-dependent susceptibility to opioid dependence.

Gene expression was first normalized using Seurat’s LogNormalize method (log_1_p-transformed counts per 10,000 total counts), and a uniform subset of 50,000 cells was randomly sampled per experimental group (AA SAL, AA MOR, GG SAL, GG MOR) to ensure balanced representation. Mean gene expression was then computed for each Allen Mouse Brain Atlas CCFv3 level-7 region, yielding a region-by-gene matrix per group. Only regions with robust sampling (≤10 missing values across genes) were retained.

Pearson correlation coefficients were calculated between all pairs of regions using the regional gene expression profiles. To construct high-confidence co-expression networks, we retained only region pairs with a correlation coefficient > 0.95. While biologically relevant co-expression relationships can occur below this stringent threshold, using a high correlation threshold (>0.95) facilitates clear visualization of robust transcriptional couplings and reduces interpretive complexity inherent in high-dimensional network analysis. Nodes in the resulting networks represented brain regions, and edges denoted significant transcriptional similarity. Node degree centrality—defined as the number of connections per region normalized to the total number of possible connections—was computed to quantify each region’s topological prominence.

To enable anatomical interpretation, brain regions were assigned parent mesostructure identities (CCFv3 level-3 acronyms) and visualized using a standardized color palette consistent across figures. Network graphs were embedded onto sagittal brain contours (z = 510) using region centroid coordinates derived from the Allen Mouse Connectivity Atlas annotation volume. This spatial projection enabled visualization of inter-regional transcriptional coupling overlaid on brain anatomy. To support visual interpretability, node sizes were scaled according to degree centrality, and edge widths were proportional to co-expression strength. Visual encoding was bounded using the 5th and 95th percentiles of the full score distribution to minimize the influence of outliers. This graph-based framework enabled direct comparison of network topology between genotypes and treatment conditions, revealing region-specific rewiring of transcriptional connectivity across the brain in opioid-naïve and morphine-exposed animals (Supplemental Fig 6).

### WGCNA Module Detection and Analysis

#### Data Preprocessing and Region-Level Expression Matrix Generation (Fig 4A)

Single-nucleus transcriptomic data were processed using Seurat (v4.3.0) to obtain normalized, log-transformed gene expression matrices. Cells were annotated with anatomical region (Allen CCFv3, “level 7” subregion), experimental group (genotype and treatment: AA SAL, AA MOR, GG SAL, GG MOR), and region meta-data. For WGCNA input, we computed mean expression for each gene within each region × experimental group combination, generating a region × gene expression matrix. Regions with fewer than 100 cells per group were excluded to minimize sampling noise. All steps were implemented in R (v4.2.3). A threshold of ≥100 cells per region was empirically selected, consistent with recommendations for robust estimation of mean gene expression in spatial and single-cell RNA-seq analyses, minimizing technical variability and ensuring statistical reliability in downstream WGCNA analysis^46,54^. Alternative thresholds produced unstable modules or insufficient statistical power.

#### Consensus (Pooled) WGCNA: Network Construction Across All Groups

For global module detection (Fig. 4b, d–k), WGCNA was performed on the pooled region × gene matrix, combining all regions and groups. Traits included region, genotype, and treatment (binary-encoded). Matrix rows corresponded to region-group pairs (e.g., “PVT_AA MOR”), and columns to genes.

Network construction followed standard WGCNA procedures^54^:

- Sample and gene filtering was performed via goodSamplesGenes.
- Soft-thresholding power for adjacency matrix construction was selected using pickSoftThreshold (powers 1–20), prioritizing scale-free topology (signed R2 > 0.8). We empirically determined power = 8 as optimal—balancing sufficient module segmentation, stable topology, and the ability to resolve biologically distinct modules. Rationale and diagnostic plots are provided (Supplementary Fig. 5A,B).
- Consensus modules were identified with blockwiseModules (minModuleSize=20, deepSplit=3, pamRespectsDendro=FALSE, mergeCutHeight=0.10, numericLabels=FALSE), using biweight midcorrelation and dynamic tree cutting. Consensus modules represent gene modules consistently co-expressed across genotype and treatment conditions, thereby identifying core transcriptional networks independent of condition-specific variation. This consensus approach enables more robust biological interpretations compared to condition-specific networks alone^19,54^.
- Module color assignments (dynamicColors) and dendrograms were saved for downstream trait correlation and module mapping.

All region-gene matrices, trait meta-data, eigengenes, and module assignments were saved for reproducibility.

#### Per-Group WGCNA: Module Detection Within Each Experimental Group

To identify modules specific to each experimental group (used for Fig. 4c and region-specific module/pathway analysis), the same WGCNA workflow was applied independently to each group (AA SAL, AA MOR, GG SAL, GG MOR):

- For each group, a region × gene matrix was generated using only cells from the relevant experiment group. Regions with <100 cells were excluded.
- Module detection parameters matched the consensus analysis (power=8, minModuleSize=20, etc.) for strict comparability across groups.
- Each group’s WGCNA output was saved separately for subsequent module–region mapping and enrichment analyses.

#### Module–Trait Correlation and Anatomical Heatmaps

To relate modules to anatomical, genotype, and treatment traits, we calculated Pearson correlations between **M**odule **E**igengenes (**ME**; the first principal component of each module which is considered a representative of the gene expression profiles in a module) and binary trait matrices:

- For region-level mapping, a one-hot encoded region matrix was constructed (regions × samples). MEs were correlated to each region indicator.
- Genotype and treatment were encoded as binary variables and correlated with MEs.
- All correlation and p-value matrices were exported for visualization (heatmaps/barplots) and interpretation **(see** Supplementary Fig. 5c).
- Regional order and color annotations followed anatomical hierarchy and AP location, as established from Allen CCFv3 meta-data.
- Strong module–trait correlations indicate that module-associated genes participate in biological processes specifically responsive to genetic background or opioid exposure, highlighting candidate mechanisms underlying vulnerability or resilience to OUD.

#### Differential Module Dynamics and Circos Visualization (Fig 4B)

To summarize module dynamics across genotype and treatment, we constructed a consensus module-level annotation table integrating the following:

- OUD risk gene enrichment (from GWAS).
- Differential expression (DEG) between groups (AA MOR vs AA SAL, GG MOR vs GG SAL, AA vs GG in SAL, AA vs GG in MOR), using paired region-wise t-tests across matched regions (FDR < 0.05, |log2FC| > 0.25).
- Enrichment for OUD risk genes and DEGs in each module was assessed by Fisher’s exact test, with negative log10-transformed p-values capped at 10.
- The resulting table was visualized as a concentric circos plot: inner rings represented enrichment for OUD risk and each DEG effect, while the outer ring corresponded to the module color. Normalized enrichment scores were visualized using diverging colormaps (Matplotlib v3.7), with module labels arranged for clarity.

#### Prioritization of Dynamic Modules

To identify modules with the most pronounced differential regulation, we ranked modules according to:

- Difference in enrichment for MOR vs SAL DEGs between genotypes (|log10_p_DEG_TREAT_AA − log10_p_DEG_TREAT_GG|).
- Highest enrichment for each contrast (OUD, treatment, genotype).

The top three modules per metric were selected for downstream network analysis.

#### Inter-Regional Differential Coexpression Networks (Fig 4 d.e.j.k)

For each prioritized module and genotype (AA, GG), we constructed inter-regional coexpression networks capturing treatment-induced reorganization (MOR vs SAL):

- For each module, expression matrices were extracted for the relevant regions and genes, separately for SAL and MOR groups.
- Region–region coexpression matrices (Pearson correlation) were calculated for each condition; the difference matrix (MOR – SAL) represented differential network connectivity.
- Edges were normalized by maximum absolute difference; only top-weighted edges (up to 200 per network) were retained for clarity. Normalization by maximum absolute correlation difference effectively highlights the most significant network rearrangements; however, it may under-represent subtle yet biologically meaningful connectivity alterations that are characteristic of transcriptional regulatory networks, a common limitation in differential network analysis.
- Node attributes included pathway enrichment (top 50 module genes per region, analyzed by clusterProfiler and org.Mm.eg.db) and cell count (Seurat meta-data).
- Networks were visualized on sagittal Allen Brain Atlas coordinates (using the AllenSDK MouseConnectivityCache), with nodes colored by top enriched pathway, and edges by normalized weight and difference.

#### Module Hub Gene and Subnetwork Analysis (Fig 4 f.g.h.i)

For each differential module network, hub gene analysis was performed:

- ARACNe/minet-based mutual information networks were inferred separately for MOR and SAL regional matrices.
- Hub genes (highest node degree) were ranked by change in hub score (Δhub = MOR – SAL).
- For each module/genotype, top three hub genes and their immediate neighbors (max 25 nodes) were visualized and exported for further interpretation. The maximum node count (25) for hub-gene subnetworks was selected to balance interpretability and complexity, consistent with standard practice in network biology visualization studies. Larger subnetworks were assessed but did not significantly enhance biological interpretation.

#### Per-Group Region–Module Mapping and GO Slim Enrichment (Fig 4c)

To identify region-specific modules, we performed module eigengene–region mapping:

- For each group, module eigengenes were averaged by region.
- Z-scores for each module’s eigengene across regions were computed; modules with |z| > 1.5 in a single region were flagged as region-specific.
- Modules enriched in opioid-relevant regions^55,56^ were selected for functional annotation.
- GO Slim parent-category mappings were computationally derived using QuickGO without subjective interventions, ensuring an objective and reproducible categorization of enriched biological processes according to established bioinformatics protocols^57^.

GO enrichment analysis (GO:BP, clusterProfiler, org.Mm.eg.db) was performed on module genes within region-specific modules (background: all expressed genes in group). Top pathways (FDR < 0.05) were mapped to GO Slim parent categories using QuickGO^57^ for interpretability. All statistical tests were rigorously corrected for multiple comparisons using stringent false discovery rate adjustments (Benjamini–Hochberg, q < 0.05) to minimize false positives^58^. While adequately powered for moderate-to-large transcriptional effects, subtle expression changes may remain statistically undetected, reflecting inherent limitations of high-throughput transcriptomic methods^59^.

#### Shared Pathway Visualization and Annotation

For comprehensive cross-group comparison, top 60 pathways (by frequency and FDR) were selected and mapped across modules, regions, and experiment groups:

- Each pathway was assigned its parent GO Slim category, with multiple mappings allowed.
- Pathways were visualized using ggplot2 (v3.4.0) as a faceted heatmap, with axes indicating region–module–group, and tiles colored by −log10(adj. p-value).
- Facets corresponded to parent GO Slim categories for biological interpretability (see Supplemental Table 1).
- All code, annotation tables, and intermediate results were version-controlled and are available upon request.

#### Limitations and future directions

Although our methods provide comprehensive and high-resolution characterization of transcriptional and cellular dynamics, inherent limitations remain, including challenges in detecting subtle transcriptional changes in rare or transcriptionally quiescent cell populations, and potential dropout effects common in single-cell and spatial transcriptomics^60^. Future methodological improvements, including increased cell sampling depth, advanced imputation algorithms, or integration with complementary omics techniques (e.g., ATAC-seq), could further elucidate subtle molecular dynamics underlying opioid dependence. Our analysis focused exclusively on female mice due to documented genotype-dependent effects being more robust in females for opioid responses^8,20^. While this maximized statistical power and biological resolution, the single-sex design inherently limits direct generalization to males, necessitating future comparative studies to determine sex-specific molecular underpinnings.

#### Software and Computational Tools

All spatial transcriptomic preprocessing, spatial registration, clustering, differential expression, and high-dimensional visualization were performed using a combination of custom and established computational pipelines in R (v4.3), Python (v3.10), and MATLAB (R2023b).

### Core Analytical Platform

#### CellDynamicST (in-house platform; to be released)

All spatial transcriptomic workflows including data preprocessing, anatomical mapping, clustering, and spatial visualization were executed using **CellDynamicST**, a unified, modular software platform developed in-house. CellDynamicST integrates both core analytical tools and custom-built modules for spatially resolved single-cell data analysis. It wraps Python, R, and MATLAB dependencies into a reproducible, container-compatible framework designed for seamless deployment across high-performance computing environments.

The platform is organized into eight major modules (see Supplementary Fig. 1) that reflect key stages of the spatial transcriptomic analysis pipeline:

- Data Registration
  - Image preprocessing and reference slice reconstruction
  - Anatomical alignment using the STSHARCQ module (a modified version of SHARCQ with enhanced single-cell mapping)
  - Visual validation of slice-to-atlas registration
- Data Preprocessing
  - Hierarchical anatomical annotation assignment (Allen Brain Atlas CCFv3, depths 3–7)
  - Experimental condition mapping and anatomical tree construction
- Cell Typing
  - Generalized unsupervised clustering with genotype- and treatment-aware correction
  - Supervised label propagation across conditions
  - Cell-type annotation using neurotransmitter identity, glial marker modules, and regional context
  - Marker gene detection and manual refinement for ambiguous clusters
- Atlas Visualization
  - Cell type and neurotransmitter mapping onto brain coordinates
  - Sagittal atlas projection (z=510) with spatial overlay and color-encoded annotations
- Spatial Cell Dynamics
  - Quantification and comparison of cell-type distributions across regions and genotypes
  - Visualization of region- and cell-specific redistributions in response to opioid dependence
- Functional Dynamics
  - Single-cell and region-level GO enrichment via weighted GSEA
  - Comparative visualization of enriched biological processes
- Connectivity Dynamics
  - Inter-regional gene co-expression networks and centrality analysis
  - Pairwise inter-regional differential gene expression (DGE) with heatmap visualizations
  - Module specific Inter-regional Differential Expressed Network Visualizations
- Specialized Applications
  - Dynamic modeling of cell-state transitions (e.g., control energy simulation in opioid dependence)
  - Cross-species neuropsychiatric risk inference using external gene sets (e.g., from AnocoDB)
  - Cell type–based vulnerability mapping using machine learning classifiers

#### Release:

Upon publication, **CellDynamicST** will be released as an open-source toolkit via GitHub and archived with a DOI. The release will include:

- Full API documentation
- Executable Jupyter and RMarkdown notebooks
- Example datasets
- Environment files for pip and Conda
- Command-line tools for batch processing

#### R Packages

The following R packages (v4.3 environment) were used for spatial transcriptomic data analysis, annotation, network construction, statistical modeling, and visualization:

Core Analysis and Modeling

- Seurat v4.3, SeuratDisk – Single-cell normalization, scaling, clustering, dimensionality reduction, and differential gene expression analysis.
- DESeq2 v1.38, limma, multcomp – Negative binomial modeling and statistical inference for inter-regional gene expression.
- DoubletFinder, TSCAN – Doublet detection and trajectory inference.
- mclust, flashClust, Matrix, matrixStats – Clustering diagnostics and matrix-level operations.

Gene Ontology and Functional Enrichment

- clusterProfiler v4.0, fgsea, pathview – Gene set enrichment analysis and pathway visualization.
- GO.db, org.Mm.eg.db, msigdbr, AnnotationDbi, biomaRt – Ontology databases and gene ID conversions for Mus musculus.

Spatial, Network, and Graph-Based Analysis

- spatialLIBD, sf – Spatial expression mapping and anatomical overlays.
- igraph, ggraph, tidygraph, networkD3, RANN, rdist, vegan, DescTools – Graph theory analysis, distance metrics, network visualization, and diversity quantification (Gini, Shannon).

Visualization and Plotting

- ggplot2, ggpubr, cowplot, patchwork, grid, gridExtra, pheatmap, ComplexHeatmap, ComplexUpset, ggVennDiagram, ggrepel, treemap – High-resolution plotting for expression, GO terms, region-level comparisons, and set intersections.
- RColorBrewer, colorspace, viridis, pals, scales, png – Perceptual color encoding and image embedding.

Data Wrangling and Utilities

- dplyr, tidyr, purrr, tibble, readr, readxl, reshape2, stringr, data.table, tools, jsonlite, lubridate – Tidy data manipulation, IO operations, and time tracking.
- S4Vectors, parallel, future, future.apply, pbapply, progress, progressr, conflicted – Parallelization, reproducibility, and runtime tracking.

#### Python Packages

All Python analyses were conducted using Python v3.10 unless otherwise specified. Packages were installed and managed via pip or conda in isolated virtual environments to ensure reproducibility. Analyses spanned data preprocessing, spatial transcriptomic analysis, deep learning, anatomical mapping, and interactive visualization.

- Core analysis and data handling:
  scanpy v1.9.3, AnnData – for single-cell transcriptomic preprocessing, normalization, and differential expression analysis;
  numpy, pandas, scipy, scikit-learn, seaborn, matplotlib, tqdm – for matrix operations, statistics, dimensionality reduction, and high-dimensional plotting;
  h5py, gzip, json, os, shutil, tempfile, traceback, sys, time, warnings – for I/O operations, data parsing, and system integration.
- Spatial transcriptomics and neuroanatomical mapping:
  allensdk v0.16+ – for anatomical annotation using the Allen Mouse Brain Atlas (CCFv3), including centroid localization and hierarchical structure mapping;
  networkx, adjustText, PIL, imageio, skimage, shapely, geopandas – for network-based spatial graph construction, overlay rendering, and structural quantification.
  search_structure_tree, methods, utils – in-house or adapted utilities for region ontology, slicing, and geometric normalization.
- Custom tools and specialty packages:
  mellon – for density estimation in cell-state space (used in regional cell-state density plots);
  palantir, numba – for latent manifold reconstruction and accelerated matrix computations;
  pkg_resources, setuptools, unittest, logging, collections, ast – for internal dependency management and debugging.

All Python analyses were performed using reproducible pipelines with strict version control. Full environment files and example notebooks will be released alongside the dataset to ensure end-to-end reproducibility.

#### MATLAB

- **STSHARCQ (Spatial Transcriptomic SHARCQ)** – MATLAB was used to implement an enhanced version of SHARCQ for anatomical registration of spatial transcriptomic sections. STSHARCQ supports single-cell resolution mapping by integrating transcriptomic coordinates with the Allen Mouse Brain Atlas (CCFv3), enabling direct structural annotation of each cell^47^. Additional enhancements include improved geometric correction, nonlinear deformation handling, and overlay visualization of anatomical and transcriptomic layers. STSHARCQ is bundled as a core module within the **CellDynamicST** platform and will be released with full documentation and example datasets.

## Supplementary Material

1 **Supplementary Fig. 1 Integrated spatial transcriptomic analysis pipeline for genotype-and treatment-specific brain profiling.** Schematic overview of the experimental and computational workflow. Adult female Oprm1 A118G mice were assigned to genotype and treatment groups, followed by standardized tissue processing, CosMx SMI-based spatial transcriptomic imaging, and multiplexed in situ hybridization. High-resolution transcriptomic data were processed, quality-controlled, and spatially registered to the Allen Mouse Brain Atlas. Downstream analysis via the CellDynamicST platform includes hierarchical cell-type annotation, region-specific clustering, single-cell and region-level differential expression, gene ontology enrichment, spatial dynamics, and co-expression network modeling. Outputs include annotated cell type maps, regional expression summaries, pathway enrichment, network topology, and comprehensive data visualizations for morphine-dependent brain plasticity. Demonstration icons are adapted from BioRender under publication license [*JE28CD35JJ*]. The CellDynamicST logo was generated using AI-based design tools (OpenAI, 2025).
**Supplemental Fig. 2 Opioid dependent brain state characters of region-specific cell types in A118G AA mice (a) or GG mice (b).** Dot heatmaps (center) show the respective cell type distribution in Allen Atlas CCFv3 regions (depth = 7) by dot size and the percentage change of opioid dependent state over naïve state by color (orange = higher, blue = lower). Regions are organized by meso-structures (depth = 3) and ordered by anterior to posterior location. Cell types are organized and grouped by meso-structural subtypes. Neurotransmitter composition in opioid naïve or opioid dependent state is annotated for each cell type (right annotation bar 1 & 2) and for each region (top annotation bar). General endogenous receptor expression composition in each cell type is annotated (left annotation bar) in naïve and dependent state. Major Cell Type composition in each region is colored and annotated (bottom annotation bar 1 & 2). Bar plots on the right showing the Shannon diversity coefficient for each cell type for both opioid naïve and opioid dependent state.
**Supplementary Fig. 3 Cross-referenced validation of annotated cell types using CellMarker2 mouse brain database.** To evaluate the biological validity and generalizability of annotated cell clusters, we cross-referenced cluster-specific marker genes with curated cell-type gene sets from the CellMarker2 database (Zhang et al., 2023; brain-specific, mouse-only). Marker genes were identified using Seurat’s FindMarkers() with strict thresholds (log₂FC > 1, adjusted P < 0.01, min.pct ≥ 0.4, q_diff ≥ 0.7), ensuring high-confidence, cluster-enriched signatures. For each cluster, we computed an overlap score reflecting the proportion of cluster marker genes matching a CellMarker2-defined gene set, and assigned a best-matching CellMarker2 identity based on maximal overlap. Bar heights indicate overlap scores per cluster, and colors reflect the identity of the best-matching CellMarker2 cell type. While canonical neuronal and glial cell types (e.g., astrocytes, microglia, dopaminergic neurons) showed strong agreement, several clusters showed low overlap, indicating potential discovery of regionally enriched or transcriptionally novel populations not yet cataloged in public databases. These results support the annotation robustness of our spatial clustering pipeline while highlighting its potential to reveal previously uncharacterized brain cell states.
**Supplementary Fig. 4 Global landscape of cell-type distribution shifts across all cell types in opioid dependence and genotype comparison.** Unfiltered t-SNE projections of all annotated cell types across four experimental comparisons: (1) *AA SAL vs. AA MOR*, (2) *GG SAL vs. GG MOR*. For each comparison, we computed a transcriptional “disproportionality score” per cell type, defined as the mean normalized expression of treatment-responsive DEGs weighted by average absolute log_2_ fold-change (|log_2_FC|). Scores were z-transformed across all cell types to highlight transcriptionally dominant populations—those expressing DEGs at significantly higher levels than expected under null distributions (see Methods). All annotated cell types are included without filtering to provide a comprehensive view of global transcriptional responsiveness. Color represents the magnitude and direction of each cell type’s disproportionality score (coolwarm scale centered at zero); label annotations indicate cell types with nonzero scores. Spatial layouts are based on precomputed t-SNE embeddings of generalized PCA components. These unfiltered plots complement the main heatmap analysis (Fig. 1 g & h) by highlighting transcriptionally engaged cell types regardless of statistical significance, including rare or spatially localized populations.
**Supplementary Fig. 5 WGCNA module detection, diagnostic plots, and anatomical trait correlation. (a)** Scale-free topology fit and mean connectivity plots for selection of soft-thresholding power in pooled WGCNA. Power 8 was chosen to optimize scale-free topology and module resolution. **(b)** Dendrogram and module color assignment for consensus (pooled) region × gene network, identifying robust coexpression modules across all brain regions and groups. **(c)** Heatmap showing Pearson correlations between module eigengenes and anatomical regions, genotype, and treatment traits. Regional annotation and ordering follow Allen CCFv3 hierarchy and anterior–posterior spatial location.
**Supplementary Fig. 6 Inter-regional OUD-related gene co-expression network showing pair-wise region to region (Allen CCFv3, depth = 7) global gene co-expression level.** Edge color showing relative co-expression strength (Dark Grey = high, White = low). Node corresponding to center of each region, which color showing meso-structural category of each region and size of each node corresponding to degree centrality of each region (size = connections with other regions).
**Supplementary Fig. 7 Differential Network Rewiring in Additional WGCNA Modules (Pink, Blue, Yellow, Magenta). (a–d)** Atlas-projected differential gene co-expression networks for selected WGCNA modules under morphine versus saline conditions. Each panel displays region-resolved rewiring patterns in one module: pink (a), blue (b), yellow (c), and magenta (d). For each genotype (AA, GG), module-level expression was averaged across Allen CCFv3 level-7 brain regions. Inter-regional co-expression networks were computed using Pearson correlation, and visualized as overlays on a sagittal Allen Brain Atlas template (z = 510). Nodes represent brain regions, colored by the most enriched GO Biological Process pathway in the saline condition. Edge width indicates baseline co-expression strength, and edge color reflects the change in correlation under morphine (Δcorrelation). Only the top 200 strongest edges are shown per network. For each module, Δhub genes were identified based on change in network centrality between SAL and MOR conditions, and their immediate subnetworks visualized. Hub genes (gold nodes) and neighbors (sky blue) illustrate how morphine reorganizes local network topology. These modules complement Fig. 4 by highlighting additional genotype-dependent network responses across astrocytic (pink), synaptic (blue), executive (yellow), and hypothalamic/metabolic (magenta) axes.

## Data Availability

CellDynamicST will be released on GitHub [googlexie.github.io/CellDynamicST] with open-source license and full documentation. Processed data, expression matrices, and metadata will be deposited in online with accession codes provided upon acceptance.

## Footnotes

Ethical Approval All animal procedures were approved by the Institutional Animal Care and Use Committee (IACUC) of the University of Pennsylvania and were conducted in accordance with the NIH Guide for the Care and Use of Laboratory Animals. Experimental protocols and housing conditions followed those previously described by Blendy et al. (e.g., *Mague et al., 2009*) and were consistent with ethical standards for vertebrate research.

## References

[1] Agrawal A. & Lynskey M. T. Are there genetic influences on addiction: evidence from family, adoption and twin studies. Addiction 103, 1069–1081 (2008). 18494843 10.1111/j.1360-0443.2008.02213.x

[2] Goldman D., Oroszi G. & Ducci F. The genetics of addictions: uncovering the genes. Nat Rev Genet 6, 521–532 (2005). 15995696 10.1038/nrg1635

[3] Zhou H. Association of OPRM1 Functional Coding Variant With Opioid Use Disorder: A Genome-Wide Association Study. JAMA Psychiatry 77, 1072–1080 (2020). 32492095 10.1001/jamapsychiatry.2020.1206 PMC7270886

[4] Gaddis N. Multi-trait genome-wide association study of opioid addiction: OPRM1 and beyond. Sci Rep 12, 16873 (2022). 36207451 10.1038/s41598-022-21003-y PMC9546890

[5] Kember R. L. Cross-ancestry meta-analysis of opioid use disorder uncovers novel loci with predominant effects in brain regions associated with addiction. Nat Neurosci 25, 1279–1287 (2022). 36171425 10.1038/s41593-022-01160-z PMC9682545

[6] Bond C. Single-nucleotide polymorphism in the human mu opioid receptor gene alters beta-endorphin binding and activity: possible implications for opiate addiction. Proc Natl Acad Sci U S A 95, 9608–9613 (1998). 9689128 10.1073/pnas.95.16.9608 PMC21386

[7] Chong R. Y. The mu-opioid receptor polymorphism A118G predicts cortisol responses to naloxone and stress. Neuropsychopharmacology 31, 204–211 (2006). 16123758 10.1038/sj.npp.1300856

[8] Mague S. D. Mouse model of OPRM1 (A118G) polymorphism has sex-specific effects on drug-mediated behavior. Proc Natl Acad Sci U S A 106, 10847–10852 (2009). 19528658 10.1073/pnas.0901800106 PMC2705603

[9] Zhang Y. Mouse model of the OPRM1 (A118G) polymorphism: differential heroin self-administration behavior compared with wild-type mice. Neuropsychopharmacology 40, 1091–1100 (2015). 25336208 10.1038/npp.2014.286 PMC4367451

[10] Mague S. D. & Blendy J. A. OPRM1 SNP (A118G): involvement in disease development, treatment response, and animal models. Drug Alcohol Depend 108, 172–182 (2010). 20074870 10.1016/j.drugalcdep.2009.12.016 PMC2887703

[11] Halikere A. Addiction associated N40D mu-opioid receptor variant modulates synaptic function in human neurons. Mol Psychiatry 25, 1406–1419 (2020). 31481756 10.1038/s41380-019-0507-0 PMC7051890

[12] Ament S. A. The single-cell opioid responses in the context of HIV (SCORCH) consortium. Mol Psychiatry 29, 3950–3961 (2024). 38879719 10.1038/s41380-024-02620-7 PMC11609103

[13] Wei J. Single nucleus transcriptomics of ventral midbrain identifies glial activation associated with chronic opioid use disorder. Nat Commun 14, 5610 (2023). 37699936 10.1038/s41467-023-41455-8 PMC10497570

[14] Avey D. Single-Cell RNA-Seq Uncovers a Robust Transcriptional Response to Morphine by Glia. Cell Rep 24, 3619–3629.e4 (2018). 30257220 10.1016/j.celrep.2018.08.080 PMC6357782

[15] Browne C. J. Transcriptional signatures of heroin intake and relapse throughout the brain reward circuitry in male mice. Sci. Adv. 9, eadg8558 (2023). 37294757 10.1126/sciadv.adg8558 PMC10256172

[16] Karagiannis T. T. Single cell transcriptomics reveals opioid usage evokes widespread suppression of antiviral gene program. Nat Commun 11, 2611 (2020). 32457298 10.1038/s41467-020-16159-y PMC7250875

[17] Phan B. N. Single nuclei transcriptomics in human and non-human primate striatum in opioid use disorder. Nat Commun 15, 878 (2024). 38296993 10.1038/s41467-024-45165-7 PMC10831093

[18] Reiner B. C. Single nucleus transcriptomic analysis of rat nucleus accumbens reveals cell type-specific patterns of gene expression associated with volitional morphine intake. Transl Psychiatry 12, 374 (2022). 36075888 10.1038/s41398-022-02135-1 PMC9458645

[19] Seney M. L. Transcriptional Alterations in Dorsolateral Prefrontal Cortex and Nucleus Accumbens Implicate Neuroinflammation and Synaptic Remodeling in Opioid Use Disorder. Biol Psychiatry 90, 550–562 (2021). 34380600 10.1016/j.biopsych.2021.06.007 PMC8463497

[20] Xie Y., Brynildsen J. K., Windisch K. & Blendy J. A. Neural Network Connectivity Following Opioid Dependence is Altered by a Common Genetic Variant in the μ-Opioid Receptor, *OPRM1* A118G. J. Neurosci. 44, e1492232023 (2024). 38124015 10.1523/JNEUROSCI.1492-23.2023 PMC10866092

[21] Hu C. CellMarker 2.0: an updated database of manually curated cell markers in human/mouse and web tools based on scRNA-seq data. Nucleic Acids Res 51, D870–D876 (2023). 36300619 10.1093/nar/gkac947 PMC9825416

[22] Mansour A., Khachaturian H., Lewis M. E., Akil H. & Watson S. J. Anatomy of CNS opioid receptors. Trends Neurosci 11, 308–314 (1988). 2465635 10.1016/0166-2236(88)90093-8

[23] Wang Y.-J., Huang P., Ung A., Blendy J. A. & Liu-Chen L.-Y. Reduced expression of the μ opioid receptor in some, but not all, brain regions in mice with OPRM1 A112G. Neuroscience 205, 178–184 (2012). 22240251 10.1016/j.neuroscience.2011.12.033 PMC3772531

[24] Jiang Y., Wang X. & Dong C. Molecular mechanisms of T helper 17 cell differentiation: Emerging roles for transcription cofactors. Adv Immunol 144, 121–153 (2019). 31699215 10.1016/bs.ai.2019.09.003

[25] Herman M. A. GIRK3 gates activation of the mesolimbic dopaminergic pathway by ethanol. Proc Natl Acad Sci U S A 112, 7091–7096 (2015). 25964320 10.1073/pnas.1416146112 PMC4460485

[26] Wang Y. & Nie G. Overview of Human HtrA Family Proteases and Their Distinctive Physiological Roles and Unique Involvement in Diseases, Especially Cancer and Pregnancy Complications. Int J Mol Sci 22, 10756 (2021). 34639128 10.3390/ijms221910756 PMC8509474

[27] Koldobskiy M. A. p53-mediated apoptosis requires inositol hexakisphosphate kinase-2. Proc Natl Acad Sci U S A 107, 20947–20951 (2010). 21078964 10.1073/pnas.1015671107 PMC3000257

[28] Duke-Cohan J. S. Attractin (DPPT-L), a member of the CUB family of cell adhesion and guidance proteins, is secreted by activated human T lymphocytes and modulates immune cell interactions. Proc Natl Acad Sci U S A 95, 11336–11341 (1998). 9736737 10.1073/pnas.95.19.11336 PMC21643

[29] Opferman J. T. & Kothari A. Anti-apoptotic BCL-2 family members in development. Cell Death Differ 25, 37–45 (2018). 29099482 10.1038/cdd.2017.170 PMC5729530

[30] Guseva D. Cell Adhesion Molecule Close Homolog of L1 (CHL1) Guides the Regrowth of Regenerating Motor Axons and Regulates Synaptic Coverage of Motor Neurons. Front Mol Neurosci 11, 174 (2018). 29881335 10.3389/fnmol.2018.00174 PMC5976800

[31] Loers G. Interplay in neural functions of cell adhesion molecule close homolog of L1 (CHL1) and Programmed Cell Death 6 (PDCD6). FASEB Bioadv 4, 43–59 (2022). 35024572 10.1096/fba.2021-00027 PMC8728108

[32] Namekata K. Dock3 stimulates axonal outgrowth via GSK-3β-mediated microtubule assembly. J Neurosci 32, 264–274 (2012). 22219288 10.1523/JNEUROSCI.4884-11.2012 PMC6621311

[33] Zhang Y. KCNQ Channels Enable Reliable Presynaptic Spiking and Synaptic Transmission at High Frequency. J Neurosci 42, 3305–3315 (2022). 35256530 10.1523/JNEUROSCI.0363-20.2022 PMC9034779

[34] Lehnardt S. The toll-like receptor TLR4 is necessary for lipopolysaccharide-induced oligodendrocyte injury in the CNS. J Neurosci 22, 2478–2486 (2002). 11923412 10.1523/JNEUROSCI.22-07-02478.2002 PMC6758325

[35] Vestweber D. & Blanks J. E. Mechanisms that regulate the function of the selectins and their ligands. Physiol Rev 79, 181–213 (1999). 9922371 10.1152/physrev.1999.79.1.181

[36] Palazuelos J., Klingener M. & Aguirre A. TGFβ signaling regulates the timing of CNS myelination by modulating oligodendrocyte progenitor cell cycle exit through SMAD3/4/FoxO1/Sp1. J Neurosci 34, 7917–7930 (2014). 24899714 10.1523/JNEUROSCI.0363-14.2014 PMC4044250

[37] Zhang Y., Randesi M., Blendy J. A., Kreek M. J. & Butelman E. R. Impact of OPRM1 (Mu-opioid Receptor Gene) A112G Polymorphism on Dual Oxycodone and Cocaine Self-administration Behavior in a Mouse Model. Neuroscience 539, 76–85 (2024). 38211933 10.1016/j.neuroscience.2024.01.002

[38] Zhang Y., Collins D., Butelman E. R., Blendy J. A. & Kreek M. J. Relapse-like behavior in a mouse model of the OPRM1 (mu-opioid receptor) A118G polymorphism: Examination with intravenous oxycodone self-administration. Neuropharmacology 181, 108351 (2020). 33031806 10.1016/j.neuropharm.2020.108351

[39] Collins D., Zhang Y., Blendy J. & Kreek M. J. Murine model of OPRM1 A118G alters oxycodone self-administration and locomotor activation, but not conditioned place preference. Neuropharmacology 167, 107864 (2020). 31778689 10.1016/j.neuropharm.2019.107864

[40] Robinson S. A., Jones A. D., Brynildsen J. K., Ehrlich M. E. & Blendy J. A. Neurobehavioral effects of neonatal opioid exposure in mice: Influence of the OPRM1 SNP. Addict Biol 25, e12806 (2020). 31267641 10.1111/adb.12806 PMC8450766

[41] Popova D., Desai N., Blendy J. A. & Pang Z. P. Synaptic Regulation by OPRM1 Variants in Reward Neurocircuitry. J Neurosci 39, 5685–5696 (2019). 31109961 10.1523/JNEUROSCI.2317-18.2019 PMC6636083

[42] Stickels R. R. Highly sensitive spatial transcriptomics at near-cellular resolution with Slide-seqV2. Nat Biotechnol 39, 313–319 (2021). 33288904 10.1038/s41587-020-0739-1 PMC8606189

[43] Maynard K. R. Transcriptome-scale spatial gene expression in the human dorsolateral prefrontal cortex. Nat Neurosci 24, 425–436 (2021). 33558695 10.1038/s41593-020-00787-0 PMC8095368

[44] Maldonado R. Reduction of Morphine Abstinence in Mice with a Mutation in the Gene Encoding CREB. Science 273, 657–659 (1996). 8662559 10.1126/science.273.5275.657

[45] Brynildsen J. K. Gene coexpression patterns predict opiate-induced brain-state transitions. Proc. Natl. Acad. Sci. U.S.A. 117, 19556–19565 (2020). 32694207 10.1073/pnas.2003601117 PMC7431093

[46] Stuart T. Comprehensive Integration of Single-Cell Data. Cell 177, 1888–1902.e21 (2019). 31178118 10.1016/j.cell.2019.05.031 PMC6687398

[47] Lauridsen K. A Semi-Automated Workflow for Brain Slice Histology Alignment, Registration, and Cell Quantification (SHARCQ). eNeuro 9, ENEURO.0483-21.2022 (2022). 10.1523/ENEURO.0483-21.2022 PMC9034756 35396257

[48] Lein E. S. Genome-wide atlas of gene expression in the adult mouse brain. Nature 445, 168–176 (2007). 17151600 10.1038/nature05453

[49] Haghverdi L., Lun A. T. L., Morgan M. D. & Marioni J. C. Batch effects in single-cell RNA-sequencing data are corrected by matching mutual nearest neighbors. Nat Biotechnol 36, 421–427 (2018). 29608177 10.1038/nbt.4091 PMC6152897

[50] Butler A., Hoffman P., Smibert P., Papalexi E. & Satija R. Integrating single-cell transcriptomic data across different conditions, technologies, and species. Nat Biotechnol 36, 411–420 (2018). 29608179 10.1038/nbt.4096 PMC6700744

[51] Yao Z. A high-resolution transcriptomic and spatial atlas of cell types in the whole mouse brain. Nature 624, 317–332 (2023). 38092916 10.1038/s41586-023-06812-z PMC10719114

[52] Hu C. CellMarker 2.0: an updated database of manually curated cell markers in human/mouse and web tools based on scRNA-seq data. Nucleic Acids Research 51, D870–D876 (2023). 36300619 10.1093/nar/gkac947 PMC9825416

[53] Knoedler J. R. A functional cellular framework for sex and estrous cycle-dependent gene expression and behavior. Cell 185, 654–671.e22 (2022). 35065713 10.1016/j.cell.2021.12.031 PMC8956134

[54] Langfelder P. & Horvath S. WGCNA: an R package for weighted correlation network analysis. BMC Bioinformatics 9, 559 (2008). 19114008 10.1186/1471-2105-9-559 PMC2631488

[55] Fields H. L. & Margolis E. B. Understanding opioid reward. Trends in Neurosciences 38, 217–225 (2015). 25637939 10.1016/j.tins.2015.01.002 PMC4385443

[56] Koob G. F. & Volkow N. D. Neurocircuitry of Addiction. Neuropsychopharmacol 35, 217–238 (2010). 10.1038/npp.2009.110 PMC2805560 19710631

[57] Binns D. QuickGO: a web-based tool for Gene Ontology searching. Bioinformatics 25, 3045–3046 (2009). 19744993 10.1093/bioinformatics/btp536 PMC2773257

[58] Benjamini Y. & Hochberg Y. Controlling the False Discovery Rate: A Practical and Powerful Approach to Multiple Testing. Journal of the Royal Statistical Society Series B: Statistical Methodology 57, 289–300 (1995).

[59] Conesa A. A survey of best practices for RNA-seq data analysis. Genome Biol 17, 13 (2016). 26813401 10.1186/s13059-016-0881-8 PMC4728800

[60] Svensson V. Droplet scRNA-seq is not zero-inflated. Nat Biotechnol 38, 147–150 (2020). 31937974 10.1038/s41587-019-0379-5

