# Supplementary material for: Spatial transcriptomics reveals distinct cell type dynamics following opioid dependence in mice with the common human variant in the μ-opioid receptor, Oprm1 A118G
Source: Res Sq. 2025 Aug 18:rs.3.rs-7199524. Preprint. [Version 1] doi: 10.21203/rs.3.rs-7199524/v1 (PMC12393594; doi:10.21203/rs.3.rs-7199524/v1)

Supplemental Fig 1

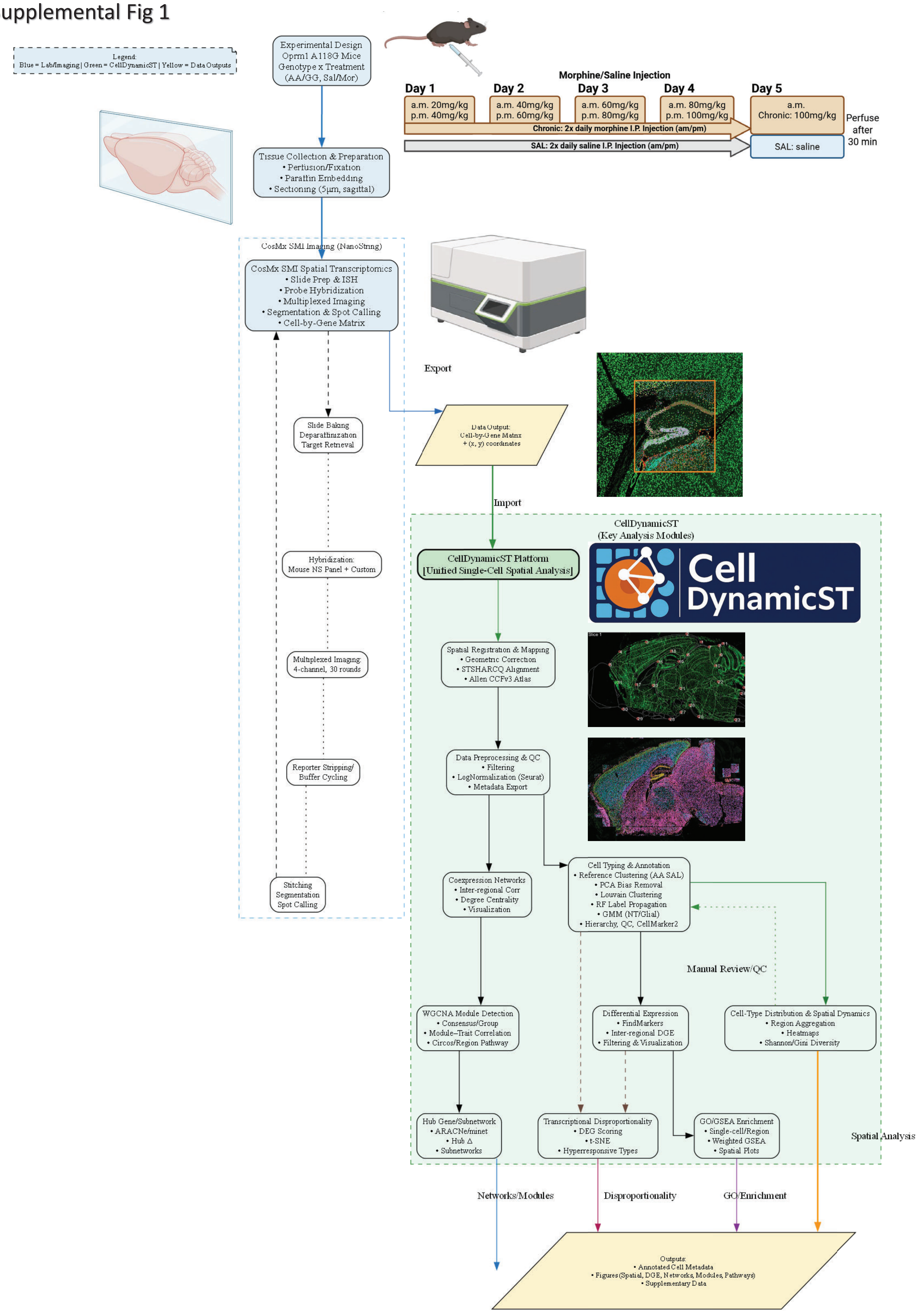

Supplemental Fig 2A

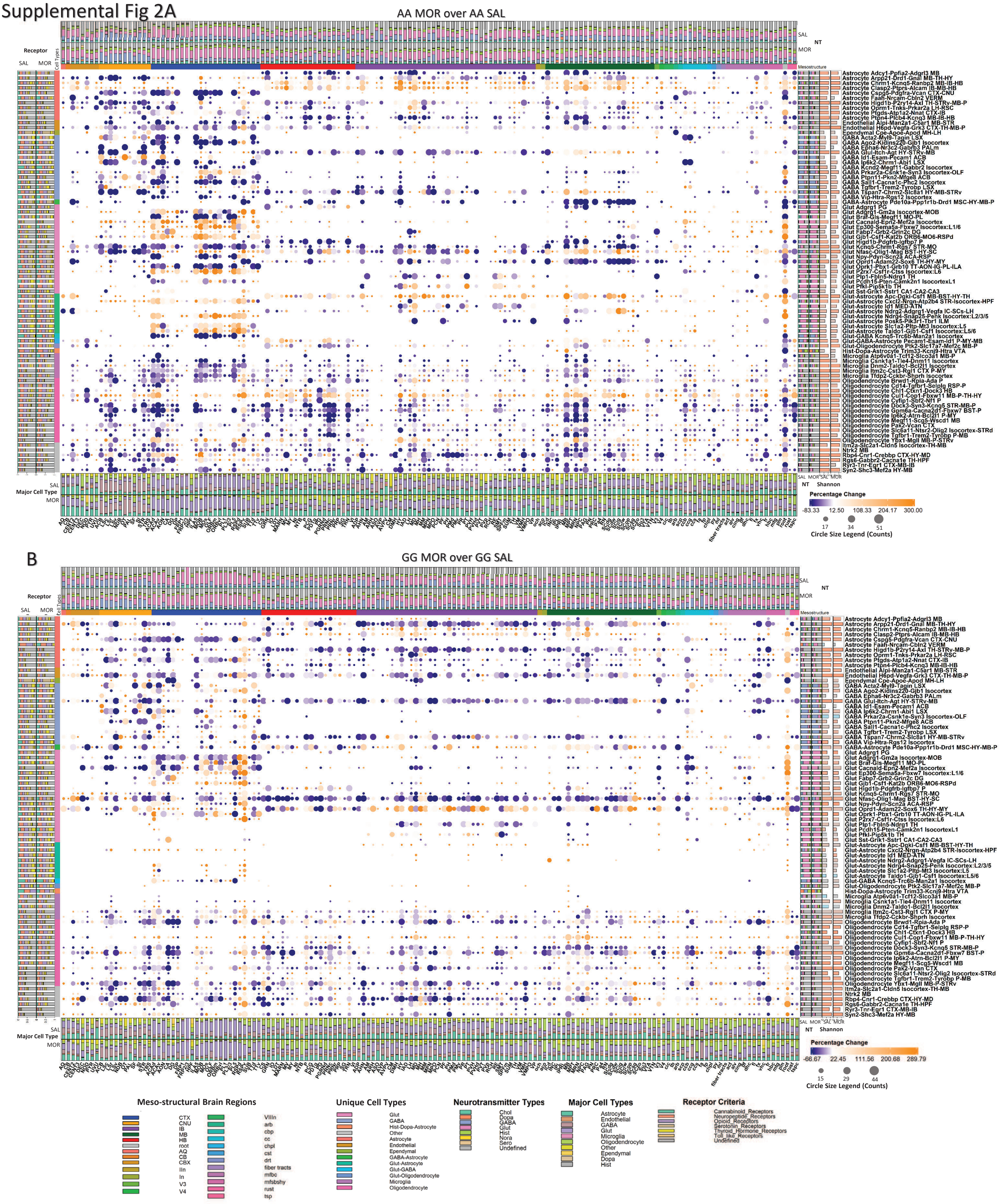

Supplemental Fig 3

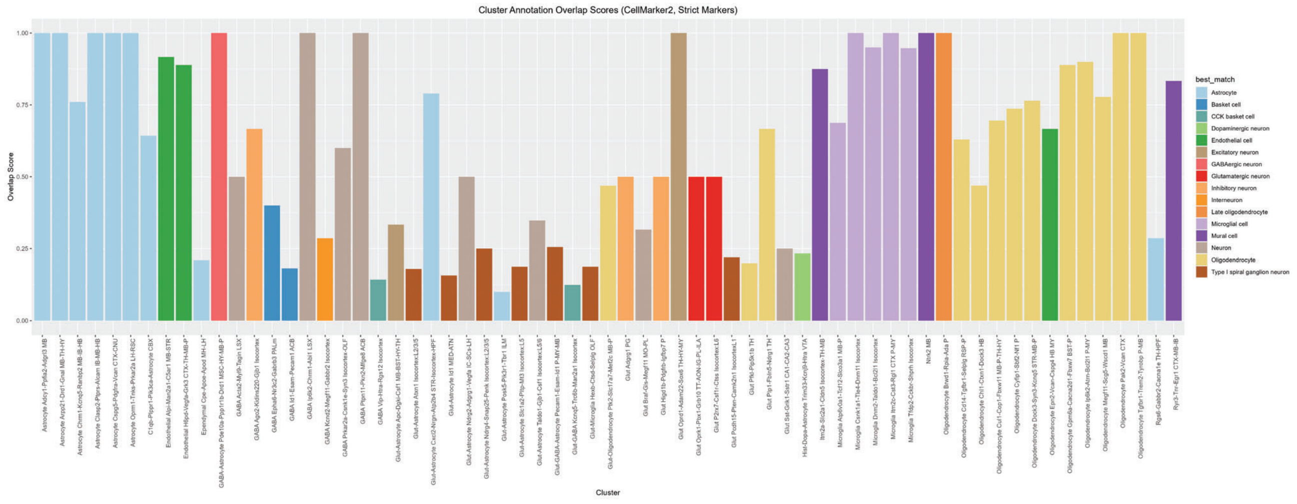

Supplemental Fig 4

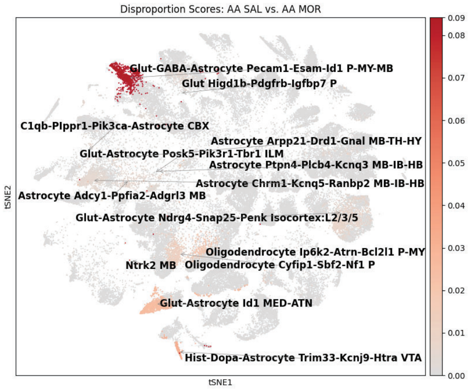

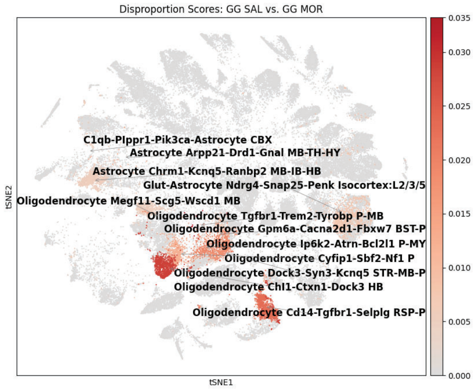

Supplemental Fig 5

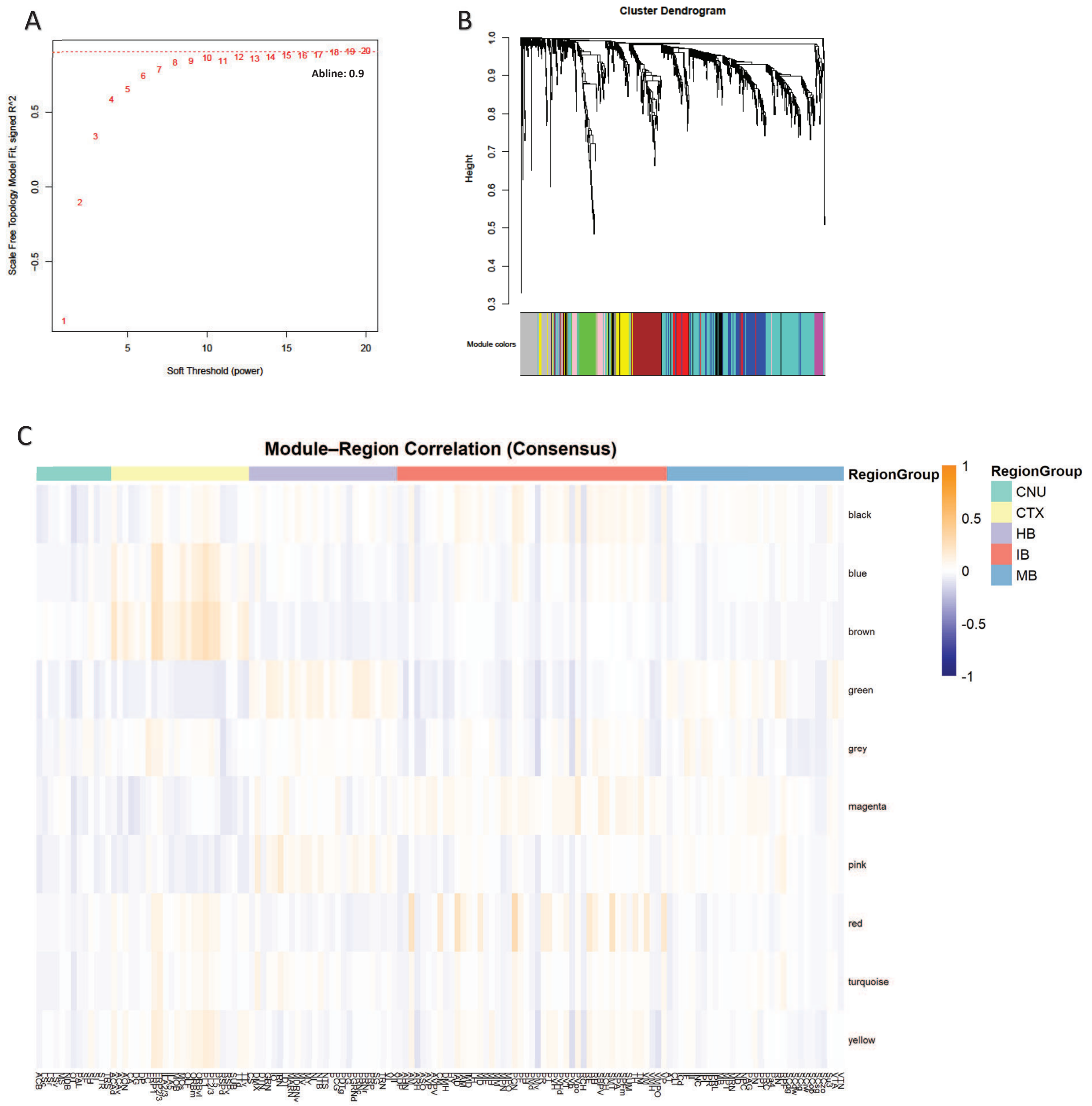

Supplemental Fig 6

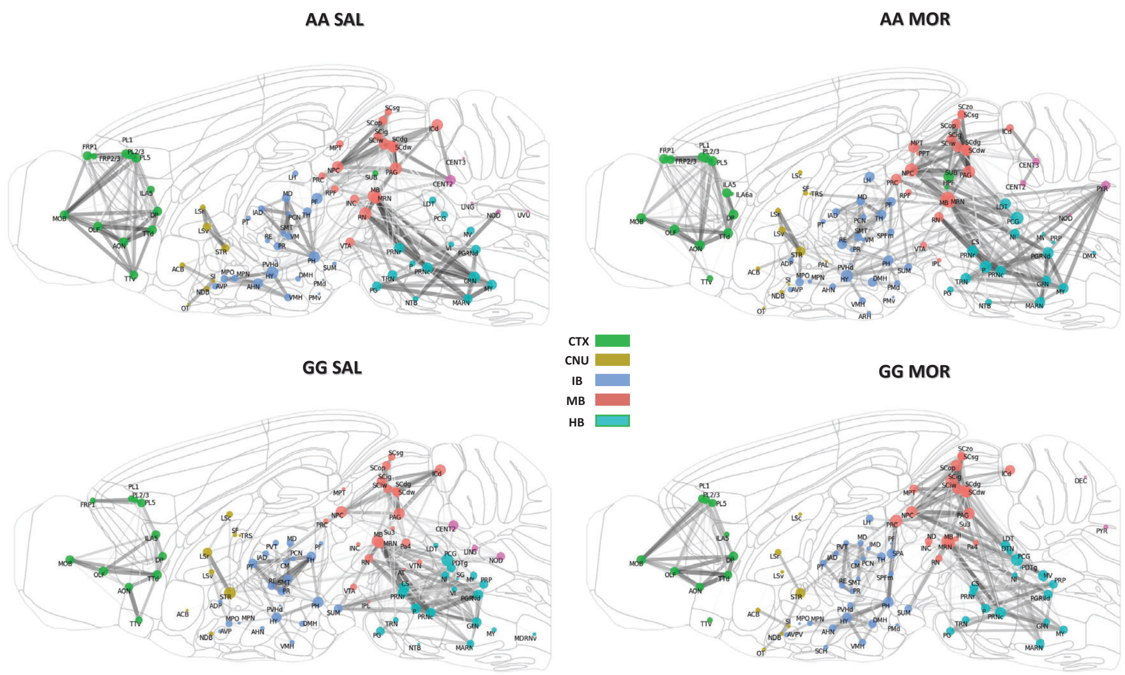

Supplemental Fig 7

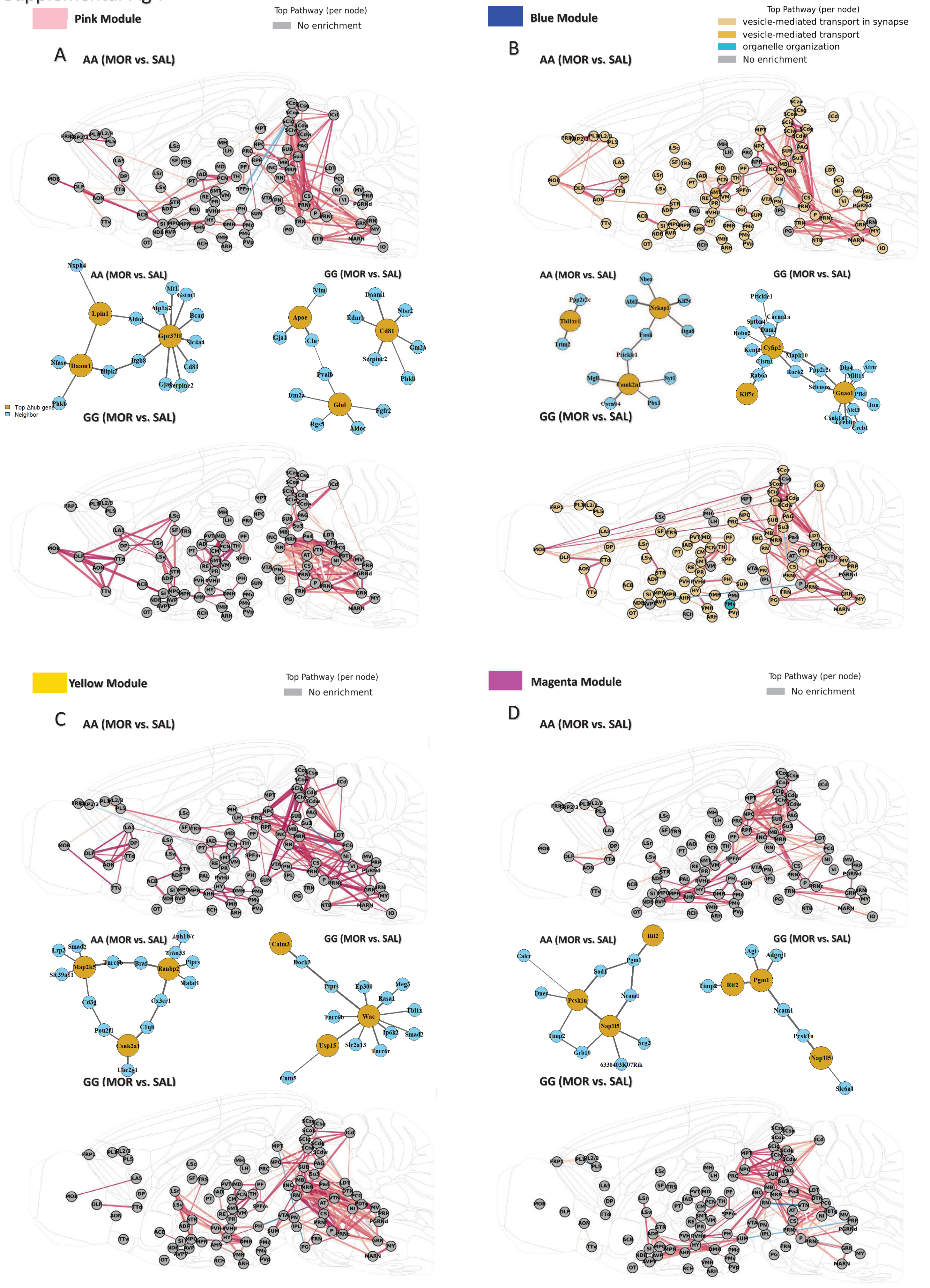

Supplement: 1 — Supplementary Fig. 1 Integrated spatial transcriptomic analysis pipeline for genotype-and treatment-specific brain profiling. Schematic overview of the experimental and computational workflow. Adult female Oprm1 A118G mice were assigned to genotype and treatment groups, followed by standardized tissue processing, CosMx SMI-based spatial transcriptomic imaging, and multiplexed in situ hybridization. High-resolution transcriptomic data were processed, quality-controlled, and spatially registered to the Allen Mouse Brain Atlas. Downstream analysis via the CellDynamicST platform includes hierarchical cell-type annotation, region-specific clustering, single-cell and region-level differential expression, gene ontology enrichment, spatial dynamics, and co-expression network modeling. Outputs include annotated cell type maps, regional expression summaries, pathway enrichment, network topology, and comprehensive data visualizations for morphine-dependent brain plasticity. Demonstration icons are adapted from BioRender under publication license [JE28CD35JJ]. The CellDynamicST logo was generated using AI-based design tools (OpenAI, 2025). Supplemental Fig. 2 Opioid dependent brain state characters of region-specific cell types in A118G AA mice (a) or GG mice (b). Dot heatmaps (center) show the respective cell type distribution in Allen Atlas CCFv3 regions (depth = 7) by dot size and the percentage change of opioid dependent state over naïve state by color (orange = higher, blue = lower). Regions are organized by meso-structures (depth = 3) and ordered by anterior to posterior location. Cell types are organized and grouped by meso-structural subtypes. Neurotransmitter composition in opioid naïve or opioid dependent state is annotated for each cell type (right annotation bar 1 & 2) and for each region (top annotation bar). General endogenous receptor expression composition in each cell type is annotated (left annotation bar) in naïve and dependent state. Major Cell Type composit [file NIHPPRS7199524V1-supplement-1.pdf]
